# Tracking of activated cTfh cells following sequential influenza vaccinations reveals transcriptional profile of clonotypes driving a vaccine-induced immune response

**DOI:** 10.3389/fimmu.2023.1133781

**Published:** 2023-03-29

**Authors:** Jennifer Currenti, Joshua Simmons, Jared Oakes, Silvana Gaudieri, Christian M. Warren, Rama Gangula, Eric Alves, Ramesh Ram, Shay Leary, Jesse D. Armitage, Rita M. Smith, Abha Chopra, Natasha B. Halasa, Mark A. Pilkinton, Spyros A. Kalams

**Affiliations:** ^1^ School of Human Sciences, University of Western Australia, Crawley, WA, Australia; ^2^ Division of Infectious Diseases, Department of Medicine, Vanderbilt University Medical Center, Nashville, TN, United States; ^3^ Department of Pathology, Microbiology, and Immunology, Vanderbilt University Medical Center, Nashville, TN, United States; ^4^ Institute for Immunology and Infectious Diseases, Murdoch University, Murdoch, WA, Australia; ^5^ Telethon Kids Institute, University of Western Australia, Nedlands, WA, Australia; ^6^ Division of Pediatric Infectious Diseases, Department of Pediatrics, Vanderbilt University Medical Center, Nashville, TN, United States

**Keywords:** influenza virus, vaccination, cTfh, TCR - T cell receptor, clonal expansion

## Abstract

**Introduction:**

A vaccine against influenza is available seasonally but is not 100% effective. A predictor of successful seroconversion in adults is an increase in activated circulating T follicular helper (cTfh) cells after vaccination. However, the impact of repeated annual vaccinations on long-term protection and seasonal vaccine efficacy remains unclear.

**Methods:**

In this study, we examined the T cell receptor (TCR) repertoire and transcriptional profile of vaccine-induced expanded cTfh cells in individuals who received sequential seasonal influenza vaccines. We measured the magnitude of cTfh and plasmablast cell activation from day 0 (d0) to d7 post-vaccination as an indicator of a vaccine response. To assess TCR diversity and T cell expansion we sorted activated and resting cTfh cells at d0 and d7 post-vaccination and performed TCR sequencing. We also single cell sorted activated and resting cTfh cells for TCR analysis and transcriptome sequencing.

**Results and discussion:**

The percent of activated cTfh cells significantly increased from d0 to d7 in each of the 2016-17 (p < 0.0001) and 2017-18 (p = 0.015) vaccine seasons with the magnitude of cTfh activation increase positively correlated with the frequency of circulating plasmablast cells in the 2016-17 (p = 0.0001) and 2017-18 (p = 0.003) seasons. At d7 post-vaccination, higher magnitudes of cTfh activation were associated with increased clonality of cTfh TCR repertoire. The TCRs from vaccine-expanded clonotypes were identified and tracked longitudinally with several TCRs found to be present in both years. The transcriptomic profile of these expanded cTfh cells at the single cell level demonstrated overrepresentation of transcripts of genes involved in the type-I interferon pathway, pathways involved in gene expression, and antigen presentation and recognition. These results identify the expansion and transcriptomic profile of vaccine-induced cTfh cells important for B cell help.

## Introduction

Influenza vaccines play a major role in preventative health, although not all individuals are able to generate protective immunity following vaccination. A reduction in the ability to generate protective immunity is largely seen in individuals who are over the age of 65, immunocompromised and/or pregnant. Such variation in vaccine efficacy rates reflects differences in the immunological capacity of individuals to respond to the same immunogen. An additional mitigating factor influencing natural and vaccine-induced immunity against influenza is antigenic drift [the accumulation of mutations in surface proteins haemagglutinin (HA) and neuraminidase (NA)] (1). Annual influenza vaccinations that attempt to compensate for these changes in the virus have long been recommended, however the impact of sequential seasonal vaccinations on long-term protection and seasonal vaccine efficacy remains unclear.

The ability of a vaccine immunogen to stimulate B cells to generate neutralizing antibodies and immunological memory are important correlates of an effective immune response. T follicular helper (Tfh) cells are a subset of CD4^+^ T cells that provide critical help to B cells in the production of antibodies. Tfh cells were originally identified in germinal centers; however, a circulating counterpart (cTfh) has been identified that express the homing chemokine receptor CXCR5^+^ on the cell surface. When activated, cTfh cells produce IL-21 and IL-10, cytokines involved in the expansion and differentiation of B cells, akin to germinal center Tfh cells (2). Furthermore, there is a clear clonotypic relationship between the Tfh and cTfh cell populations, as shown by the overlap in the TCRβ repertoire of tonsillar Tfh cells and peripheral cTfh cells in healthy subjects (3). Accordingly, cTfh cells can be used as an accessible cell population to assess the role of Tfh cells in pathogen-specific immune responses.

In a vaccine setting, there is a direct positive correlation between the increase in the magnitude of cTfh cell activation and influenza-induced antibody production (4–8). Furthermore, cTfh cells isolated from influenza vaccine recipients have been shown to preferentially differentiate B cells and stimulate influenza-specific antibody secretion over other CD4^+^ T cell subsets (4). These studies provide strong support that cTfh cells play a critical role in influenza vaccine-induced responses. However, few studies have attempted to determine the cTfh clonotypes that may be driving a vaccine-induced response and track their frequency across vaccination seasons. Furthermore, little is known of the transcriptional profile of vaccine-induced cTfh cells.

The ability to identify and track T cell clonotypes across vaccination seasons is dependent on characterizing the TCR repertoire. The TCR repertoire allows recognition of a substantial array of T cell epitopes (9) and has been shown to be a critical component of pathogen-specific immune responses (10, 11). Furthermore, the level of TCR repertoire diversity has been implicated in the outcomes of several infectious diseases. For example, decreased TCR repertoire diversity in subjects with TB has been associated with disease severity (10), while increased virus-specific TCR repertoire diversity is associated with decreased HIV-1 variation (12) and improved Ebola disease outcome (13). Cross-reactive T cells, those able to recognize different antigens with the same TCR, have also been implicated in the variability of disease outcome following infection with influenza virus and severe acute respiratory syndrome coronavirus 2 (SARS-CoV-2) (14–18).

While TCR diversity and cross-reactivity can be beneficial upon infection with new pathogens, the TCR repertoire of the pool of memory T cells is important for recognition of antigenically similar viruses, as occurs with seasonal influenza virus infections. The magnitude of an individual’s memory CD4^+^ T cell response is negatively correlated with the severity of symptoms and viral shedding after challenge with influenza strains (17). Herati et al. showed that sequential seasonal influenza vaccinations induce a recurrent oligoclonal TCR repertoire response associated with an increase in activated cTfh cells following vaccination (19). While this study was able to identify re-stimulated memory cTfh cell responses after sequential influenza vaccinations, the impact of re-stimulating memory responses on vaccination outcome or the transcriptional profile of these memory cells was not identified. Consequently, there is a need to determine the relationship between memory cTfh responses and other measures of effective vaccine-induced immune responses following sequential influenza vaccination.

We longitudinally followed healthy individuals after sequential seasonal influenza vaccinations to track and characterize cTfh clonotypes driving vaccine-induced immune responses across seasons. Expanded cTfh cell populations following vaccination were identified in several individuals using high-resolution TCR repertoire analysis. Single cell transcriptomic analysis of these expanded cTfh cells identified several pathways involved in IFN signalling, IL-21 expression, and the regulation of gene expression and responses to stress. This study will aid in the identification of transcriptional profiles characteristic of an effective vaccine-induced cellular immune response.

## Results

To examine the role of cTfh cells in influenza vaccine responses, we evaluated 40 healthy individuals who received sequential influenza vaccinations in the USA 2016-17 and 2017-18 seasons with quadrivalent split virus vaccines. Subjects were between 23 and 68.2 (median of 35.8) years at first enrolment (see **Table 1** for cohort demographics) and peripheral blood mononuclear cells (PBMC) were isolated at timepoints day (d)0, d7 and d28 post vaccination for each season. The influenza A (H1N1 and H3N2) and B strains used for the 2016-17 and 2017-18 seasons are shown in **Table 2** and indicates only the influenza A H1N1 strain differs between the two vaccine seasons, with similar vaccine effectiveness observed across seasons (20, 21); vaccine effectiveness was determined using a logistic regression model contrasting the odds of confirmed influenza cases by RT-PCR to negative cases amongst patients seeking outpatient care for acute respiratory illness (20, 21). Influenza vaccination was mandatory for these individuals as they were healthcare workers, and as such, it is likely they also received the 2015-16 vaccine the season prior to study enrolment, albeit unknown.

**Table 1:** Subject demographics.

	All subjects	Subjects with TCR sequencing
**N**	40	12
Age (years)*	35.8 (30.6-46.2)	27.3 (25.3-30.5)
**Sex (male)**	13 (32.5)	5 (41.7)
Race		
**Asian**	2 (5)	1 (8.3)
**Black or African American**	5 (12.5)	3 (25)
**White**	32 (80)	7 (58.3)
**Other/mixed**	1 (2.5)	1 (8.3)

Numbers are n (%) and *median (interquartile range; IQR).

**Table 2:** Components and efficacy of seasonal influenza vaccines (USA).

Year/Season	Vaccine Components*				Vaccine Effectiveness (%)^#^
	H1N1 (Subtype A)	H3N2 (Subtype A)	Subtype B (Yamagata lineage)	Subtype B (Victoria lineage)	
2015-16	A/California/7/2009	A/Switzerland/9715293/2013	B/Phuket/3073/2013	-	48
2016-17	A/California/7/2009	A/Hong Kong/4801/2014	B/Phuket/3073/2013	B/Brisbane/60/2008	40
2017-18	A/Michigan/45/2015^$^	A/Hong Kong/4801/2014	B/Phuket/3073/2013	B/Brisbane/60/2008	38
2018-19	A/Michigan/45/2015^$^	A/Singapore/INFIMH-16-0019/2016	B/Phuket/3073/2013	B/Colorado/06/2017	29
2019-20	A/Brisbane/02/2018^$^	A/Kansas/14/2017	B/Phuket/3073/2013	B/Colorado/06/2017	39
2020-21	A/Guangdong-Maonan/SWL1536/2019^$^	A/HongKong/2671/2019	B/Phuket/3073/2013	B/Washington/02/2019	-
2021-22	A/Victoria/2570/2019^$^	A/Cambodia/e0826360/2020	B/Phuket/3073/2013	B/Washington/02/2019	-

^*^FDA; ^#^US CDC; ^$^pdmo9-like. - indicates no data available (vaccine effectiveness was not calculated for the 2020-21 vaccine due to low influenza virus circulation).

### cTfh activation and plasmablast frequency increase from d0 to d7 following influenza vaccination

**Figure 1:**
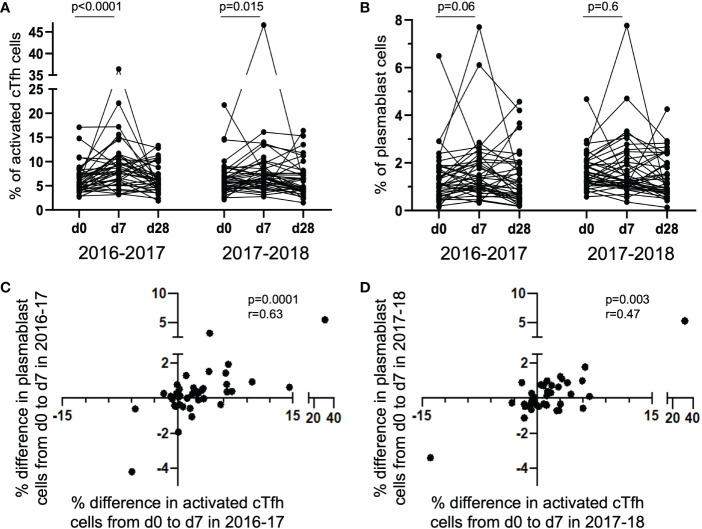
Activated cTfh and plasmablast cells increase from d0 to d7 with the magnitude of responses correlated between the cell types. The percentage of activated cTfh cells significantly increased at d7 following vaccination in the 2016-17 and 2017-18 seasons [**(A)** paired t-test], but only a trend was observed for plasmablast cells in the 2016-17 season [**(B)**; paired t-test]. The magnitude of cTfh activation and circulating plasmablast frequency is significantly positively correlated in both the 2016-17 [**(C)**; p < 0.0001; r_s_ = 0.63; Spearman Rank test] and 2017-18 [**(D)**; p = 0.003; r_s_ = 0.47; Spearman Rank test] seasons.

The degree of change in the frequency of activated cTfh cells from d0 to d7 has been shown to predict the magnitude of subsequent antibody titers following influenza vaccination (4, 5, 8). To determine if this was observed in the study cohort, we measured the magnitude of vaccine-induced cTfh cell (CD4^+^CXCR5^+^PD-1^+^) activation in the 40 subjects at timepoints d0, d7 and d28; cTfh cell activation was defined as dual expression of surface CD38 and ICOS [cTfhCD38^+^ICOS^+^ (8, 19, 22)]. A significant increase in the percentage of activated cTfh cells was observed from d0 to d7 in both 2016-17 and 2017-18 vaccination seasons (**Figure 1A**). However, there was only a trend for an increase in the frequency of circulating plasmablasts (CD19^+^CD27^+^CD38^+^) from d0 to d7 in the 2016-17 vaccination season (**Figure 1B**). When the magnitude of change from d0 to d7 for both activated cTfh and plasmablast cells were considered, a significant relationship between the two cell types was observed in both seasons (**Figures 1C, D**). These results are consistent with previous work from our group (8) and that of others (4–7). No correlation was observed between the magnitude of change for activated cTfh or plasmablast cells from d0 to d7 between seasons among individuals (**Supplementary Figure 1**). It should be noted that the cTfh and plasmablast frequencies we observed represent cumulative responses to any or all the vaccine components.

### Haemagglutinin inhibition titers following influenza vaccination are dependent on strain-specific baseline titers but not cTfh activation and plasmablast frequency

In the same 40 individuals, HAI titers to the H1N1 strains present in the 2016-17 (A/California/7/2009) and 2017-18 (A/Michigan/45/2015) vaccines and B/Phuket/3073/2013 (present in both seasonal vaccines) were determined for each year, with a 4-fold increase in HAI titers from d0 to d7 considered significant and titers above 40 considered protective (23). Despite the high positive degree of correlation between the degree of cTfh activation and the frequency of circulating plasmablasts after vaccination, for both seasons, we found no correlation between the magnitude (or fold change) of the cTfh cell response and fold increase in HAI titers for the H1N1 or B/Phuket/3073/2013 immunogen (Spearman rank correlation).

**Figure 2:**
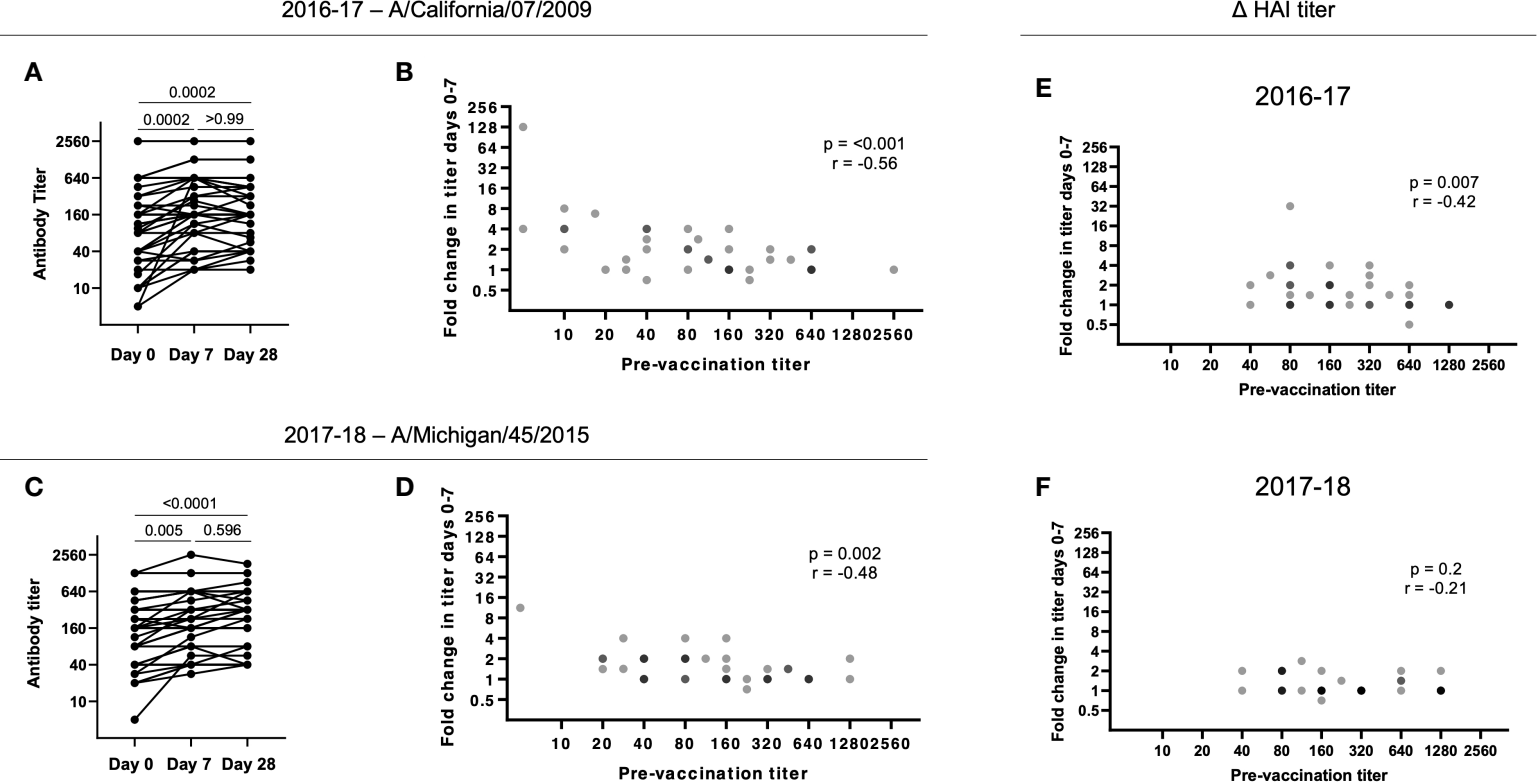
Pre-existing HAI titers impact antibody responses following vaccination. **(A)** HAI titers to A/California/7/2009 significantly increase from d0 to d7 in 2016-17 (p = 0.0002) with no significant decrease at d28 (p > 0.99;Friedman paired test with Dunn’s multiple comparisons correction). **(B)** The HAI titer fold change from d0 to d7 for A/California/7/2009 is negatively correlated with pre-vaccination (d0) titer in 2016-17 (p < 0.001; Spearman rank correlation). **(C)** HAI titers to A/Michigan/45/2015 significantly increase from d0 to d7 in 2017-18 (p = 0.005) with no significant decrease at d28 (p = 0.60; Friedman paired test with Dunn’s multiple comparisons correction). **(D)** The HAI titer fold change from d0 to d7 for A/Michigan/45/2015 is negatively correlated with pre-vaccination (d0) titer in 2017-18 (p = 0.002; Spearman rank correlation). The HAI titer fold change from d0 to d7 for B/Phuket/3073/2013 is negatively correlated with pre-vaccination (d0) titer in 2016-17 (**E**; p < 0.001; Spearman rank correlation), but not in 2017-18 **(F)**.

For the H1N1 strain A/California/7/2009 included in the 2016-17 seasonal vaccine, there was a significant increase in HAI titers from d0 to d7 that was largely maintained to d28 (p = 0.0002; **Figure 2A**). However, high pre-vaccination (d0) titers resulted in a low fold change in HAI titers from d0 to d7 (p = 0.005; **Figure 2B**). Similarly, for the A/Michigan/45/2015 immunogen included in the 2017-18 seasonal vaccine, there was a significant and maintained increase in HAI titres from d0 to d7 (p < 0.0001; **Figure 2C**). Furthermore, a significant negative correlation was observed between the fold change in HAI titer from d0 to d7 and the pre-vaccination (d0) titer (p = 0.002; **Figure 2D**).

A negative impact of pre-existing influenza-specific HAI titers on the subsequent fold increase in HAI titers has been reported by others (24–26). B/Phuket/3073/2013 was included as an immunogen in 2015-16 through to 2021-22 (**Table 2**), resulting in these subjects being exposed to the same strain annually. While we found a similar negative correlation between baseline and fold increase in titers during the 2016-17 season (p = 0.007, r = -0.24), during the 2017-18 season, titers were higher at baseline, and overall changes in titers were diminished (**Figures 2E, F**). Overall, these results suggest that continuous re-exposure to the same/similar antigen will increase HAI titers if they are low but result in a diminished boost in antibody (poor serologic) response if high baseline titers are present, possibly due to antigen interference (27–29). This has recently been shown for SARS-CoV-2 vaccination, where pre-existing antibody titers negatively correlated with antibody titers following vaccination (29).

### Increased baseline HAI titers are associated with subsequent diminished responses

**Figure 3:**
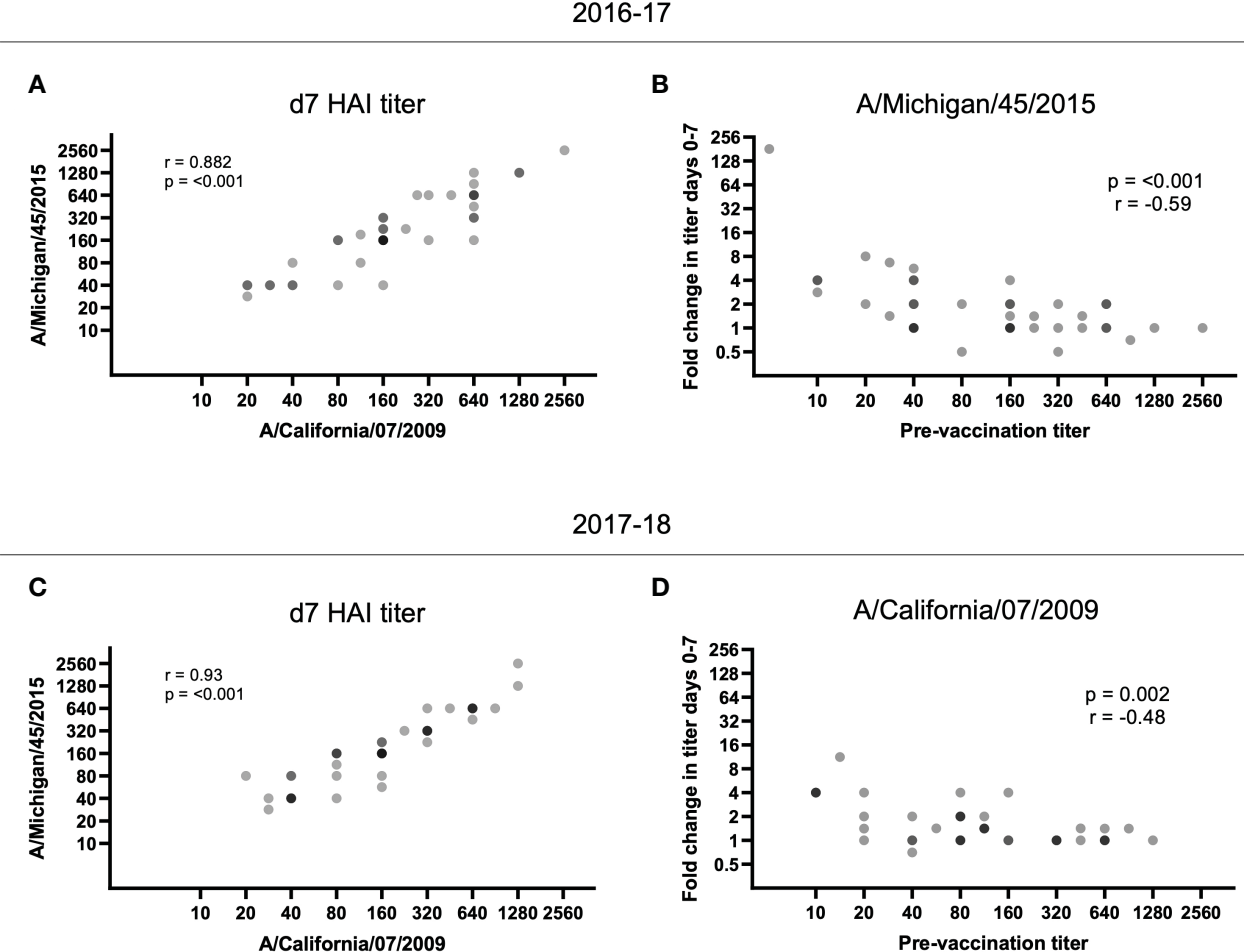
HAI titer responses to A/Michigan/45/2015 and A/California/07/2009 were identified in both vaccination seasons. The d7 HAI titer to the H1N1 A/California/7/2009 and A/Michigan/45/2015 immunogens were significantly correlated following the **(A)** 2016-17 and **(C)** 2017-18 vaccines (p < 0.001; Spearman rank correlation). **(B)** Despite no A/Michigan/45/2015 immunogen in the 2016-17 vaccine, the HAI titer fold change from d0 to d7 for A/Michigan/45/2015 is negatively correlated with pre-vaccination (d0) titer (p < 0.001; Spearman rank correlation). **(D)** Similarly, the HAI titer fold change from d0 to d7 for A/California/7/2009 is negatively correlated with pre-vaccination (d0) titer (p = 0.002; Spearman rank correlation) despite the immunogen not being present in the 2017-18 vaccine.

As we observed reduced fold increases in individuals with higher baseline titers, we next measured whether vaccination with the 2016-17 H1N1 vaccine immunogens could boost responses to the strain in use the following year. For the 2016-17 plasma, pre-existing antibodies to the A/Michigan/45/2015 strain used in the 2017-18 vaccine were present at d0 (32 participants had HAI titers of 40 or above at d0; data not shown). Furthermore, there was a significant positive correlation between the d7 HAI titer for A/California/7/2009 and A/Michigan/45/2015 for the 2016-17 plasma (p < 0.001; **Figure 3A**) and the fold change of A/Michigan/45/2015-specific antibody responses again negatively correlated with pre-vaccination titer (p < 0.0001; **Figure 3B**). Utilizing plasma from the 2017-18 vaccination season, a positive correlation was again observed between the d7 HAI titer for A/California/7/2009 and A/Michigan/45/2015 (p < 0.001; **Figure 3C**) and pre-vaccination titers negatively affected the fold change of A/California/7/2009 HAI titers from d0 to d7 (p = 0.002; **Figure 3D**). This demonstrates cross-reactive antibody responses between H1N1 antigens. Unfortunately, the available H3N2 antigen, which was shared among vaccine formulations between seasons, did not have sufficient HA activity and as such could not be included in this analysis.

### Limited differences in predicted T cell targets for immunogens across seasons

We next determined potential cross-reactivity of T cell target epitopes between the 2016-17 and 2017-18 H1N1 immunogens (**Table 2**). We determined target epitopes across the H1N1 vaccine strains based on each individual’s human leukocyte antigen (HLA) class II alleles (HLA-DR and -DQ; molecules that restrict epitope presentation to CD4^+^ T cells, including cTfh cells). Amino acid sequences for HA and NA for H1N1 strains A/California/7/2009 and A/Michigan/45/2015 were assessed for peptides with predicted binding capacity for the HLA class II alleles present in the cohort. It is important to note that the two H1N1 strains were highly similar, with 96.82% and 97.01% similarity for HA and NA, respectively. While there were several examples of peptides with different predicted binding capacity between the two H1N1 strains, the large sequence similarity likely explains the high portion of putative binding peptides that were shared between the two strains (ranging from 24-93 putative binding peptides; **Supplementary Figure 2**). However, overall, there were significantly fewer peptides with predicted binding capacity for the 2017-18 H1N1 strain and this appeared to be associated with the predicted repertoire of HLA-DR-restricted peptides (**Supplementary Figure 3**). While we were unable to detect any correlation between the number of predicted peptides with binding capacity, and magnitude of cTfh cell activation or HAI titers, the potential change in peptide repertoire may impact overall 2017-18 vaccine responses by some other mechanism. Still, these results support the previous data suggesting significant overlap in T cell targets across vaccination seasons. Identifying and validating specific influenza peptides driving cTfh cell responses is required to confirm any impact of cross-reactive T cell target epitopes on cTfh cell response and HAI titers.

### TCR diversity is inversely correlated with cTfh cell activation following vaccination, supporting a vaccine-induced clonal expansion

**Figure 4:**
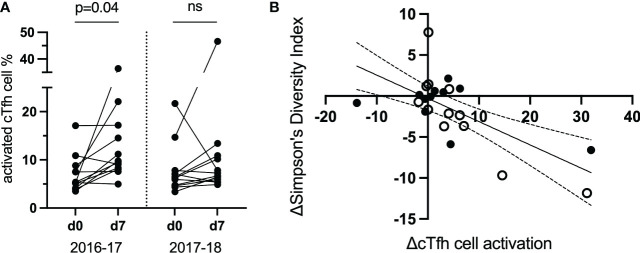
The magnitude of the increase in activated d7 cTfh cells is correlated with changes in TCR diversity. **(A)** cTfh cell activation patterns of 12 subjects selected for bulk sorting and TCRβ sequencing. **(B)**, cTfh cell activation and ,Inverse Simpson’s Index were negatively correlated (p = 0.015, r_s_ = -0.51, 95% CIs; Spearman Rank test). Values for the 2016-17 vaccine season are depicted by an open circle and for the 2017-18 vaccine season with a filled circle. ns, not significant.

TCR repertoire was assessed within and between vaccination seasons. Twelve study participants (from the original 40; **Table 1**) were selected to be examined further based on the magnitude of cTfh cell activation from d0 to d7 across the two vaccination seasons (an increase of ≥5% in cTfh cell activation in at least one season; **Figure 4A**). Activated and resting cTfh cells from these individuals were bulk sorted at d0 and d7 and subjected to TCRβ sequencing. TCR diversity was calculated using the Inverse Simpson’s Index for all cell populations at all timepoints. The degree of cTfh activation at d7 after vaccination was strongly negatively correlated with the reduction in TCR repertoire diversity of activated cTfh cells between d0 and d7 (p = 0.015; Spearman Rank test; **Figure 4B**). Of the 464,975 unique TCRβ chains identified across the 12 study participants, 529 TCRβ chains spanning all participants in both resting and activated cTfh populations have previously been reported as influenza specific (29). This observation suggests that a high degree of cTfh cell activation after vaccination reflects T cell clonal expansion.

### T cell clonotypes that expanded from d0 to d7 following vaccination can be identified across vaccination season

**Figure 5:**
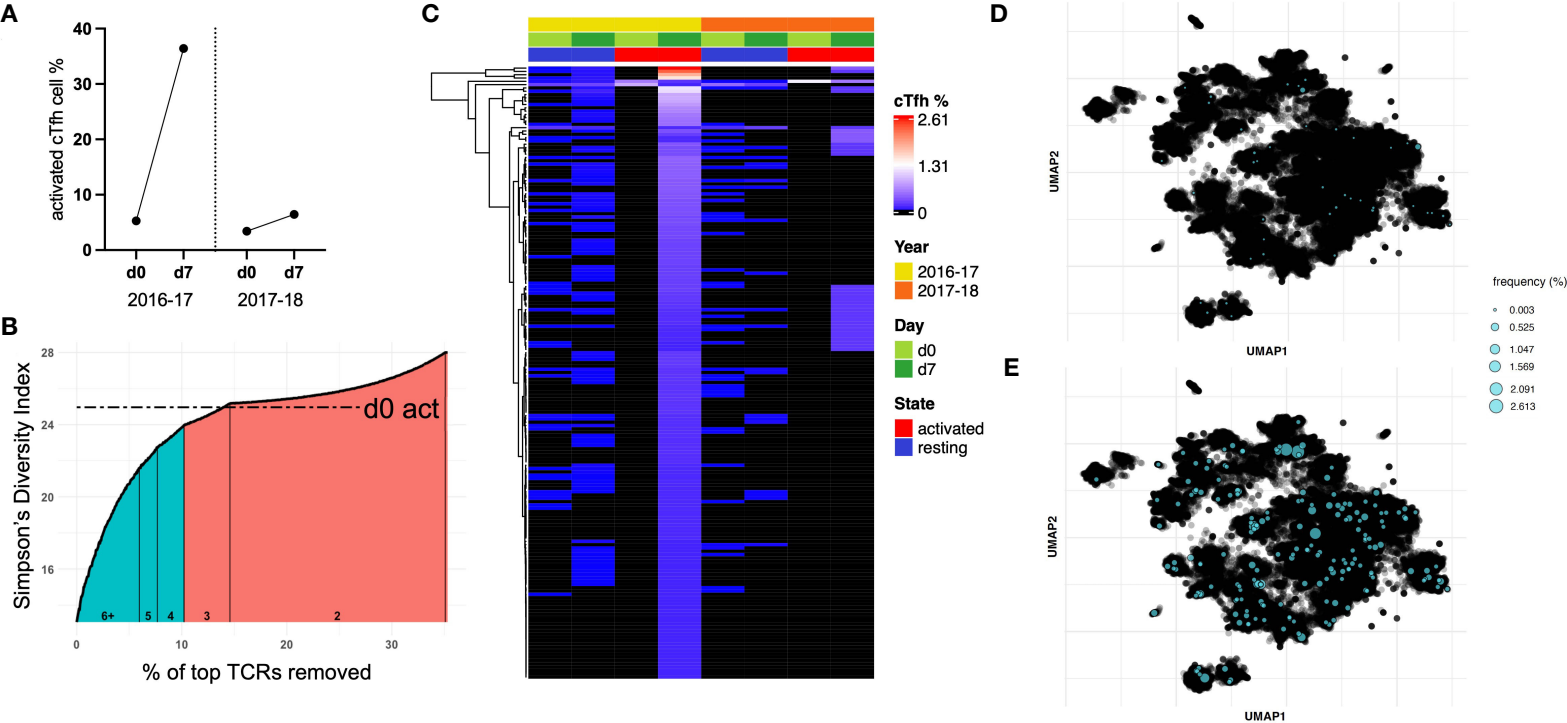
A sub-population of cTfh cells is expanded following influenza vaccination in 2016-17 and can be found after the following seasonal vaccine. **(A)** Subject 6 showed a large cTfh cell response after the 2016-17 vaccine, but only a small response was observed after the following seasonal vaccine. **(B)** Approximately 10% of activated cTfh cells at d7 expanded based on their TCR repertoire from d0 to d7 following the 2016-17 vaccine (shaded teal). As the intersection with d0 activated cTfh cell diversity fell within a count bracket, TCRs observed at this count (of 3) were not included in those deemed to be expanded. **(C)** The frequencies of cTfh cells with a TCR designated to be expanded from **(B)** are depicted for all populations as a heatmap (hierarchical clustering). Expanded cTfh cells after the 2016-17 vaccine were observed at both d0 and d7 in both years. Few expanded cTfh cells following the 2016-17 vaccine were found in the d0 active population, with 34 observed in the d7 active population after the subsequent vaccine. TCR repertoire was clustered and expanded clonotypes post-vaccination in 2016-17 were highlighted for **(D)** d0 resting and **(E)** d7 active populations.

Of the 12 study participants with bulk cTfh cell sorting, nine were identified to have a decrease in TCR diversity after vaccination. We sought to identify expanded T cell clonotypes responsible for the observed decrease in TCR diversity in these subjects and track them in the alternate vaccine season. Subject 6 demonstrated a decrease in TCR diversity from d0 to d7 in activated cTfh cells following the 2016-17 vaccine. This individual showed a high magnitude cTfh cell response following the 2016-17 vaccination (33.11%), compared to a smaller response the following season (3.04%; **Figure 5A**). This subject also demonstrated a 128-fold increase in HAI titers for H1N1 A/California/7/2009 following the 2016-17 influenza vaccination, but no significant change in titers to A/Michigan/45/2015 in the 2017-18 influenza vaccination. From here, we identified vaccine-induced expanded T cell clonotypes.

We used the following approach to determine expanded T cell clonotypes from d0 to d7 within the activated cTfh populations: first, the Inverse Simpson’s Index was calculated inclusive of all TCRs; second, T cell clonotypes were removed in a stepwise fashion starting with the highest frequency, recalculating and plotting the Inverse Simpson’s Index after each TCR removal (**Figure 5B**). This process was utilized to identify the point at which the diversity score intersects with that of d0 activated cTfh cells, the basal level of activation for a participant. This process split the activated cTfh cell clonotypes at d7 into expanded (those before the intersection point) and non-expanded (those after the intersection point) populations.

The frequencies of expanded cTfh cell clonotypes for subject 6 are shown in **Figure 5C** for all four populations spanning both vaccination seasons. The presence of specific T cell clonotypes across populations suggests several characteristics of expanded cTfh cells: i) a large portion are present as resting memory cTfh cells prior to and post-vaccination due to their presence in the resting populations; ii) they are likely to be vaccine-induced and not in response to another pathogen or bystander activation as only two activated cTfh cells with the relevant TCR were observed in d0 activated cell populations; and iii) only a subset of expanded cTfh cells in 2016-17 were re-stimulated following the 2017-18 vaccine, shown by the limited overlap with d7 activated cTfh cells following the 2017-18 vaccination (albeit there was a minimal cTfh cell response in 2017-18). A similar pattern can be observed for subject 10 who had a vaccine induced response following the 2017-18 vaccination only, with a decrease in TCR diversity from d0 to d7 (**Supplementary Figure 4**).

We applied deepTCR (30) to cluster the TCR repertoire of subject 6. As this methodology has been utilized to isolate antigen-specific TCRs (30), we sought to identify shared antigen targets of expanded cTfh cell clonotypes. UMAP clustering of the TCR repertoire for subject 6 identified several clusters. Mapping of expanded clonotypes in both d0 resting (**Figure 5D**) and d7 active (**Figure 5E**) populations revealed they were present in multiple clusters and at low frequency in the resting population prior to expansion post-vaccination. These results signify that expanded TCR clonotypes likely recognize multiple vaccine antigens. While clustering was somewhat driven by CDR3 length, when TCRs of the dominant length (15 amino acids) were clustered, distinct clusters were still formed and expanded TCRs remained spread across multiple clusters, still suggestive of multiple antigen targets (**Supplementary Figure 5**).

### Activated cTfh cells expanded in response to influenza vaccination display a transcriptional profile representative of a vaccine-induced cTfh cell response

Previous studies following influenza vaccination have identified whole blood protein and transcriptional differences pre- and post-vaccination (2, 19, 31, 32). In this study, we sought to identify transcriptomic changes of vaccine-induced cTfh cells (those activated cTfh cells that increased in frequency, or expanded, from d0 to d7; termed expanded cTfh cells herein) at the single cell level. From the 12 study participants selected for bulk TCRβ sequencing, four were further selected for single cell TCRα/β and RNA sequencing based on their cTfh cell responses (activated cTfh cell increase > 5%) and a decrease in diversity for activated cTfh cells from d0 to d7 (**Supplementary Figure 6**). To identify a transcriptional profile of vaccine-induced cTfh cells, only activated cTfh cells shown to have expanded were compared to all resting cTfh cells at d7. Due to the lack of overlap in TCR repertoire of expanded cTfh cells in other populations (**Figure 5C**), we believe that these represent vaccine-induced cTfh cells.

**Figure 6:**
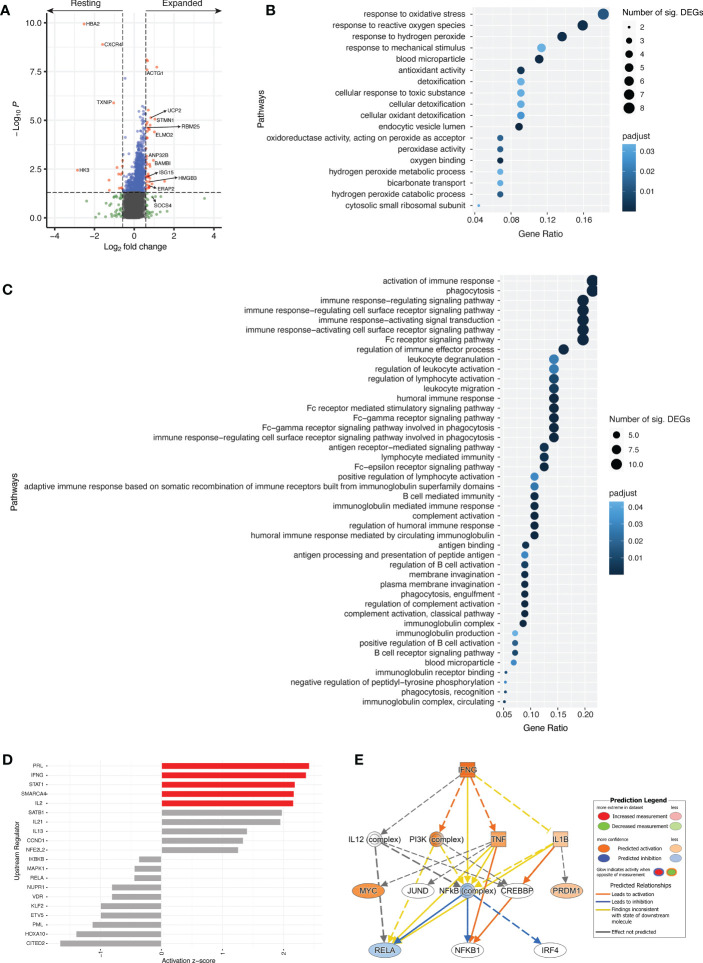
Different transcriptional profile between resting and expanded cTfh cells. **(A)** Volcano plot comparing resting and expanded cTfh cells for all four subjects at d7. Significant DEGs by both p value and fold change are depicted in red. Horizontal and vertical dotted lines represent a significance threshold of p < 0.05 and a fold change of |1.5|, respectively. Significantly upregulated GO pathways for **(B)** resting and **(C)** expanded cTfh cells. Both the number of significant DEGs within each pathway and the p adjusted value are plotted. **(D)** Top 10 upstream regulators identified using DEGs for both resting (negative) and expanded (positive) cTfh cells, with pathways demonstrating significant alteration (z-score > |2|) depicted in red. **(E)** Mechanistic network for IFNG and their activation state in expanded cTfh cells based on ingenuity pathway analysis.

Transcriptomic analyses between resting and expanded cTfh cells at d7 revealed 58 differentially expressed genes (DEGs) with p < 0.05 and a fold change greater than |1.5| (**Figure 6A**, **Table S1**). DEGs upregulated in expanded cTfh cells included genes involved in several pathways such as type-I IFN signalling (*ISG15*), gene expression/processing (*BAMBI, RBM25*), and cell processes/cytoskeletal structural elements (*ELMO2, STMN1, ACTG1, UCP2, ANP32B, HMGB3*). Transcriptomic data was not available for d0 populations, not permitting the comparison of d0 and d7 transcriptomic profiles. However, we performed transcriptomic analyses between expanded and non-expanded activated cTfh cells at d7 to determine if expanded cTfh cells had a unique profile linked to vaccination. This analysis revealed 33 DEGs with p < 0.05 and a fold change greater than |1.5| (**Supplementary Figure 7A**, **Table S2**). To further explore if the transcriptome of expanded cTfh cells is likely due to vaccination, we sought to identify where the initial significant DEGs upregulated in expanded cTfh cells (n = 43; **Table S1**) fell in this new comparison. Seven genes were significantly upregulated in expanded cTfh cells for both comparisons, with an additional 4 and 10 significant by fold change or p value alone, respectively (**Supplementary Figure 7B**). Of the remaining 22 genes, only 3 had a higher fold change for non-expanded cTfh cells (**Supplementary Figure 7C**). These results further suggest that recently (vaccine) activated cells have a unique transcriptional profile.

Genes with a fold change greater than |1.5| were subjected to pathway analyses (N = 113) (33, 34). Of the candidate genes identified, 107 were annotated with GO terms. An overrepresentation test using GO pathways identified 18 significantly overrepresented pathways for resting cTfh cells (**Figure 6B**; **Table S3**), and 44 significantly overrepresented pathways for expanded cTfh cells (**Figure 6C**; **Table S4**). These pathways were driven by 12 and 24 genes in the resting or expanded cTfh cells, respectively. For pathways upregulated in expanded cTfh cells, genes included *ACTG1, ELMO2, HMGB3*, and *SOCS4* (**Table S4**). Although several pathways associated with FcR signalling and phagocytosis were identified as significant, the genes driving these pathways overlap with those for immune activation and signalling. Furthermore, several of these genes were identified following YF19D vaccination (35). While differences in the transcriptomic expression of resting versus expanded activated cTfh cells following influenza vaccination has not been explored in depth, several of these pathways are upregulated in the transcriptome of whole blood seven days after yellow fever (35) and influenza (36) vaccination.

Upstream regulator analysis on DEGs in resting or expanded cTfh cells (log_2_FC > |1|) (37) was utilized to identify predicted drivers of the transcriptomic response between resting and expanded cTfh cells. The top 10 upstream regulators in resting (-) or expanded (+) cTfh cells were identified (**Figure 6D**). In expanded cTfh cells, five pathways were predicted to be significantly altered (activation z-score > |2|), including the canonical T cell pathways IFNG, STAT1 and IL2. Interrogation of the IFNG mechanistic network (the second strongest canonical upstream regulator) revealed a predicted downstream activation of PI3K and TNF (**Figure 6E**).

## Discussion

Antibody production is a major correlate of vaccine efficacy and is reliant on the activation of cTfh cells, however, understanding the extent and mechanisms of vaccine-induced cTfh cell activation is incomplete. Identifying a transcriptional signature of an effective vaccine-induced response would help interpretation of current vaccine strategies and potentially inform vaccine design. Numerous studies have attempted to identify a ‘universal signature’ through a systems analyses approach [transcriptional profile of whole blood, sometimes termed ‘systems vaccinology’ (38)] that can be harnessed to predict vaccine-induced immunity (39). However, the common finding among these studies is a variation in transcriptomic profiles for different vaccines (36, 39). As cTfh cells are known to aid in vaccine responses, identifying a transcriptional signature for this cell subtype can allow for specific targeting of individuals with poor vaccine responses for either different vaccination regimens, or for trials exploring novel vaccine formulations. We were able to identify a subset of vaccine-induced expanded cTfh cells with a distinct transcriptional profile. Our results provide insight into the constituents of an effective vaccine-induced immune response.

### Vaccine-specific cTfh cell responses were observed following influenza vaccination

The ability of a vaccine to activate cTfh cells is proportional to the subsequent protective antibody response (4, 5, 8), with increased expression of ICOS on cTfh cells associated with influenza-specific antibody production (2, 31, 32). We identified a significant increase in the percentage of activated cTfh cells seven days after vaccination, the magnitude of which positively correlated with circulating plasmablast frequency. While we did not observe a correlation between changes in frequency of activated cTfh cells and HAI titers, as we and others have shown (4, 5, 8), we were only able to quantify titers for the H1N1 and B strains. It is possible that cTfh activation during these years correlated with H3N2 titers, as not all immunogens are equal between seasons (40). Furthermore, higher baseline titers were observed in the 2017-18 vaccination season which may have influenced HAI titers for this season; the use of an assay permitting the correlation of absolute difference and cTfh cell activation may also be of benefit. A high fold change in HAI titers tended to result in a subsequent decrease following the next seasonal vaccination when either repeated strains were used, or when high pre-existing titers were present. These results are supported by those of others, where pre-existing antibodies can negatively impact the fold increase in HAI titers after influenza vaccination (24–26). The mechanism behind the negative impact of pre-existing antibodies is thought to be related to pre-existing influenza-specific CD4^+^ T cells capable of inhibiting antigen presentation by dendritic cells and subsequently reducing CD4^+^ T cell help to B cells (25). These findings potentially explain reduced antibody responses following repeated use of immunogens (or antigenically similar immunogens) in sequential vaccines and is supported by the large overlap of peptides with predicted binding capacity between H1N1 strains. Furthermore, this cohort comprised of younger adults who have been shown to have higher rates of seroconversion and likely higher baseline HAI titers (8).

### Clonal expansion of cTfh TCR repertoire following influenza vaccination

A previous study identified a significant positive correlation between the fold change in cTfh TCR repertoire clonality and the fold change in activated cTfh cell frequency from d0 to d7 for six subjects (19). This is consistent with our study of 12 individuals over two vaccination seasons; a decrease in diversity (increase in clonality) was observed with an increase in the proportion of activated cTfh cells, implying a clonal expansion of vaccine-specific cTfh cells in response to vaccine immunogens. Tracking these clonally expanded cTfh cells showed they were predominantly vaccine-specific (due to the lack of presence in the d0 active populations) and that they represent re-stimulated memory cTfh cells due to their presence at low levels in resting populations. These results suggest that while the re-stimulation of memory cTfh cells may be beneficial for a vaccine-induced immune response, re-stimulating the same memory cTfh cells each season is not a requirement for a corresponding serological response.

### Memory cTfh cells expand following sequential influenza vaccines

Herati et al. demonstrated repeated expansions of oligoclonal vaccine-induced cTfh in six subjects followed over at least two influenza vaccine seasons, despite changes in vaccine immunogen composition or possible intercurrent influenza infection (19). Likewise, our study demonstrated repeated expansion of the same cTfh cells following sequential influenza vaccines. It is unknown whether repeated activation of cTfh cell clonotypes represents recognition of identical epitopes or cross-reactivity to related epitopes. Re-stimulation of the same clonotypes by sequential influenza vaccines could reflect “imprinting” (or “original antigenic sin”) (41, 42), which is the inclination of vaccines to induce memory immune cells primed to respond to previous infection or vaccines to preferentially expand rather than recruit new strain-specific vaccine responses (17, 43). It is also likely that re-stimulation of the same clonotypes by sequential vaccinations is due to the large number of overlapping peptides we identified with putative binding capacity. Nonetheless, it is not yet clear if re-stimulating a pre-existing memory response (cross-reactive or not) is beneficial in a vaccine setting. We observed there was minimal overlap in the vaccine-induced cTfh TCR repertoire following sequential vaccines where only the H1N1 strain differed. Furthermore, in conjunction with the observation of a reduction in HAI titers following a previously high fold change, the use of repeated strains in influenza vaccines may reduce an individual’s ability to mount an effective vaccine-induced response.

### Expanded cTfh cells reveal potential transcriptomic signature of a successful vaccine-induced response

Differences between activated and resting cTfh cells have largely been characterized using cell surface markers, with the activation markers ICOS, CD25, CD27, CD28, CD38, CTLA4, PD-1, Helios, and Ki67 shown to have higher surface expression on activated compared to resting cTfh cells following influenza vaccination (8, 19). While characterization of cTfh cells following influenza vaccination has occurred at the cell surface level and through systems analyses, little is known about transcriptomic differences for individual cell subtypes. A comparison of the transcriptomic profile of resting versus expanded cTfh cells following influenza vaccination revealed a number of significant DEGs, several of which are associated with IFN signalling, T and B cell differentiation and proliferation, pro-inflammatory cytokine expression, gene expression/processing, and cell processes/cell cytoskeletal structural elements.

Type-I and -II IFNs regulate host anti-viral responses by inducing hundreds of IFN-stimulated genes (ISGs) and are upregulated following live attenuated yellow fever vaccine 17D (YF17D) (35) and other vaccines (35, 44), including in the transcriptome of whole blood following influenza vaccination (36). In the context of this study, several mechanistic networks were identified involving IFNs and associated pathways (PRL, IFNG, STAT1, SMARCA4, TNF, IL-2, and IL-21). Prolactin (PRL) acts to promote IFNγ secretion by T cells (45, 46) [with PRL levels positively correlated with the number of B and T cells (47, 48)], resulting in the activation of T and NK cells and the JAK/STAT signalling pathway [through STAT1 (49)]. ISGs (such as *ISG15)*, were significantly higher expressed in expanded cTfh cells. Paired with IL-21, expanded cTfh cells following influenza vaccination resemble responses to natural viral infections with impacts on T and B cell maturation and function (7, 50, 51). Additionally, genes involved in cell processes and cytoskeletal structural elements were upregulated in activated cTfh cells compared to resting cells following influenza vaccination: ELMO2, ACTG1, STMN1, ANP32B, UCP2, and HMGB3. While changes in cytoskeletal structural elements are commonly associated with tumorigenesis, the cytoskeleton also plays a role in the regulation of cellular processes such as proliferation, cell growth, and apoptosis (52), important features of a robust cellular response. The upregulation of ERAP2, an aminopeptidase that heterodimerizes and homodimerizes with ERAP1 to trim peptides prior to HLA class I loading in the endoplasmic reticulum (53) as well as shaping the cytokine response (54), highlights the upregulation of genes encoding proteins involved in antigen processing and presentation following influenza vaccination and also following YF17D vaccination (35).

cTfh cells can be divided into three subtypes based on similarities with canonical CD4^+^ T cell subpopulations: Th1-like (CXCR3^+^CCR6^-^); Th2-like (CXCR3^-^CCR6^-^); and Th17-like (CXCR3^-^CCR6^+^) (2, 4). Th2- and Th17-like cTfh cells provide better help *in vitro* to B cells (2, 31), with a transcriptional profile better reflecting that of germinal center Tfh cells (31). Based on current research, it is not clear which cTfh cell subtype would be most beneficial for producing a strong neutralizing antibody response following vaccination. However, BAMBI, PRL, and IL-2 have been shown to impact the dominant subtype of a CD4^+^ T cell response, a mechanism that could be harnessed in vaccine design once the role of cTfh subtypes has been elucidated. Unfortunately, our study was unable to identify sufficient relevant transcripts to determine cTfh cell subtype ratios. Increased expression of genes associated with cTfh subtypes, IFN responses, and anti-viral immune responses in expanded cTfh cells following influenza vaccination may provide further insight into the transcriptional profile of a vaccine-induced cTfh cell response likely to be capable of aiding B cells in the production of neutralizing antibodies.

This study confirms our prior study where we observed a strong positive correlation between post vaccination activated cTfh cells and the frequency of circulating plasmablasts; however, in contrast to our prior study (8) we did not see a correlation between increased frequency of activated cTfh cells and post vaccination antibody titers. Here, we evaluated repeat vaccination with standard dose vaccine in a cohort of individuals with a maximum age of 46. In our prior study, all individuals were older than 65, and although a significant number of individuals, responded to standard dose vaccine, cTfh activation, plasmablast frequency and the magnitude of HAI antibody titers were significantly higher in individuals receiving high dose vaccine, and this group drove the relationship between cTfh activation and fold increase in antibody titers (8). High dose vaccine induces a superior magnitude of immune responses (8) and superior protection form symptomatic illness (55). It is unclear whether this additional protection is a result of superior humoral, cellular immune responses, or both, but further study may identify additional individuals who would benefit from more immunogenic vaccine formulations.

While we were able to identify cTfh clonotypes responding to initial as well as sequential vaccination, ongoing experiments will allow us to define whether the TCRs of these cells recognize identical epitopes or are directed against recognized but altered epitopes. This is an important avenue of research that will help inform vaccine immunogen selection. There is an urgent need to improve the efficacy of influenza vaccines. For this to be achieved, the constituents of an effective vaccine-induced immune response need to be further understood to allow them to be harnessed. Clarifying the impact of cross-reactive and/or re-stimulated memory cTfh cells in sequential influenza vaccinations will inform seasonal vaccine immunogen selection. If re-stimulating memory cTfh cells is not beneficial, using the same or antigenically similar strains to the previous year may be detrimental to overall vaccine efficacy.

The identification of biomarkers to predict vaccine-induced immunity would allow adjuvants that modulate the immune response (or cTfh cell subtype ratio) to be harnessed/developed. Recent studies have aimed to identify such biomarkers in the form of a transcriptional atlas of the human immune response to several vaccinations (56, 57). While such studies have identified features of a vaccine induced immune response, they were heterogeneous at both the individual and vaccine level (56, 57). To combat this, Hagan et al. (57) proposed the development of a ‘vaccine chip’ devised to monitor plasmablast signatures as a biomarker to predict protective immune responses in vaccine trials. Due to the crucial role cTfh cells play in antibody production, we propose that the exploitation of these cells in vaccine design has the potential to not only improve the efficacy of influenza vaccines, but also inform vaccine design for many mutable pathogens.

## Materials and methods

### Study subjects

Forty healthy healthcare workers who underwent standard dose influenza vaccination for both the 2016-17 and 2017-18 seasons (see **Table 2** for vaccine components) were recruited from Vanderbilt University Medical Center (**Table 1**). Whole blood was taken at d0, d7 and d28 post-vaccination. PBMCs were purified from whole blood by FICOLL density gradient separation and cryopreserved. Four-digit resolution HLA typing (class I and class II) was performed as previously described (58).

### Ethics statement

This study was approved by the Vanderbilt Investigational Review Board and all participants provided written informed consent (Immune Responses to Respiratory Virus Vaccines, IRB 161647).

### Flow cytometry and sorting

Cryopreserved PBMCs from d0, d7 and d28 were thawed for cTfh and plasmablast analyses. PBMCs were thawed in RPMI supplemented with 10% human AB serum, 10mM glutamine and 10mM HEPES. Briefly, cells were washed and stained with CCR7-BV421 at 37°c for 15 minutes (if undergoing sorting) followed by Live/Dead Fixable Aqua (ThermoFisher) and the respective panel of fluorescently labelled antibodies (see below) for 15 minutes at room temperature.

#### cTfh panel

CD3-Alexa Fluor 700, CD4-BV605, CD8-APCy7, CD38-APC, CD27-PE-Cy7, CD19-PE-TxR, PD1-PE, ICOS-PcPCy5.5 and CXCR5-AF488 (BD Biosciences).

#### Plasmablast panel

CD3-PB, CD19-BV711, CD20-APC/AF647, CD27-PE and CD38-PE-Cy7 (BD Biosciences).

#### cTfh sorting panel

CD3-Alexa Fluor 700, CD4-BV605, CD8-APCy7, CD38-APC, TIGIT-PE-Cy7, CD45RO-PECF594, CCR7-BV421, PD1-PE, ICOS-PcPCy5.5 and CXCR5-AF488 (BD Biosciences).

Cells were washed twice and run/sorted on a BD FACS Aria III. Samples from 12 study participants were selected based on the magnitude of their cTfh response from d0 to d7 to undergo bulk sorting, with samples from four of these participants also undergoing single cell sorting. Sorted cells had 100U/mL of RNaseOUT added prior to sorting. Bulk sorted cells were centrifuged with the supernatant removed prior to freezing at -80°C. Data were analysed with FlowJo 10.1 (TreeStar). Activated and resting cTfh cells (CD4^+^CXCR5^+^PD-1^+^) were defined as CD38^+^ICOS^+^ and CD38^-^ICOS^-^, respectively (19, 22). Activated plasmablasts were defined as CD19^+^CD27^+^CD38^+^. An example of flow gating for activation and sorting is depicted in **Supplementary Figure 8**.

### Epitope binding prediction

Amino acid sequences for HA and NA for H1N1 strains A/California/7/2009 and A/Michigan/45/2015 were assessed to identify peptides with predicted binding capacity for all HLA class II alleles present in the cohort. To determine differences in the binding strength of predicted epitopes, overlapping sequences spanning 15 amino acids were inputted into the online tool NetMHCIIpan (59) to predict epitopes (of length 15) and their binding strength to a specific HLA class II allele of the HLA-DR and -DQ loci.

### HAI assay

HAI titers were assessed for all 40 study participants. Briefly, subject plasma was tested in parallel against the vaccine strains in serial doubling dilutions, with an initial dilution of 1:10. Plasma was subjected to receptor destroying enzyme and hemadsorption with 1:1 volume ratio of 0.5% turkey erythrocytes. Dilution endpoint was 1:2560; if titer exceeded the dilution series the timepoint was repeated with an endpoint of 1:20480. Subject plasma was incubated for 30 minutes at room temperature with 4 HA units/25μL of each vaccine test strain. Test strains were A/California/7/2009, A/Michigan/45/2015, and B/Phuket/3073/2013. Turkey erythrocytes were then added prior to a second 30-minute incubation. If antibodies specific for the test strain were present in the plasma, they would prevent hemagglutination of the erythrocytes. Titers were recorded as the reciprocal of the highest dilution showing complete agglutination. Titers below 10 were undetectable and represented as a titer of 5. While sera is more commonly used in HAI assays, plasma and sera have been shown to provide comparable HAI titers (60).

### Bulk TCRβ sequencing

Bulk TCRβ sequencing was undertaken on 12 study participants for both the 2016-17 and 2017-18 vaccination seasons for the d0 and d7 populations using the Adaptive Biotechnologies hsTCRb kit (Adaptive Biotechnologies, Seattle, Washington). Briefly, genomic DNA was extracted from the bulk sorted cells using DNA IQ (Promega). A multiplex PCR-based method was used to target and amplify multiple TCR targets with amplification bias accounted for with internal templates (61). Resultant templates were sequenced on the Illumina MiSeq, performed by the Vanderbilt VANTAGE DNA sequencing core. Sequences were analysed using the ImmunoSEQ platform (Adaptive Biotechnologies) and RStudio (62). TCRβ sequencing was performed at Survey level resolution (i.e. at a lower sequencing depth that is optimal for samples with low numbers of T cells and more clonal samples). Only TCR sequences unique to each subject at the nucleotide level were included in the final analyses. This conservative approach was utilized as it was not possible to distinguish between public clonotypes or contamination by rare TCR sequences. Numbers of unique and total TCRβ sequences for each subject and population are presented in **Table S5**.

### Single cell TCR and RNA sequencing

Single cell TCR and RNA sequencing was performed on samples from four subjects. Sequencing methodology was as previously described (63). Briefly, single sorted cells underwent cDNA conversion with indexed well-specific primers followed by a first round PCR with barcoded IS cDNA Amp primers. Wells were then split, and a second round PCR was conducted with either TCR or gene specific primers. Samples were then sequenced on an Illumina MiSeq using a 2x300bp paired-end chemistry kit or 600V3 kit for TCR and RNA sequencing, respectively. Reads were quality-filtered and demultiplexed prior to processing in the R package “Seurat” v4.0.3 (64–67), accession number PRJNA928207. For a gene to be included in the analysis, there were at least three cells with a count above zero for that gene. For a cell to be included in the analysis, it had to have a count above zero for more than 200 genes, between 500 - 20,000 reads, a mitochondrial content <10%, and a ribosomal content <50%. Raw data was then log normalized and scaled with the percentage of mitochondrial and ribosomal genes regressed out. Median read counts (IQR) and averages are presented in **Table S6**.

### TCR repertoire analysis

For subjects with bulk TCR sequencing, TCR diversity was calculated using the Inverse Simpson’s Index from the immunarch:::inverse_simpson function (68). Diversity scores range from 1 (no diversity, or complete clonality) to the size of the sequence pool (maximum diversity, or minimum clonality). To compensate for the range in number of TCRs sequenced for each sample, a random sampling of TCRs (equal to the lowest number of TCRs sequenced across all samples) was performed to calculate diversity, with the final score being the average of 5000 re-samplings (without replacement). By 5000, the Inverse Simpson’s Index stablilized, indicating an accurate representation of the TCR diversity for each population (see **Supplementary Figure 9** for a representative plot).

In subjects where there was an increase in clonality (i.e. decrease in diversity) from d0 to d7 in activated cTfh cells, TCRs that were driving this increase were identified using the following steps: i) TCRs were ordered by frequency (most frequent to least); ii) diversity was calculated as above; iii) the TCR with the highest frequency was removed; and iv) steps ii and iii were repeated until the diversity score of the d7 activated cell population was equal to that of the d0 activated cell population for the same vaccination season. This process splits the d7 activated cTfh cells into two groups: 1) those that expanded at d7 in comparison to d0 activated cells (cells “driving” a change in clonality after vaccination); and 2) those that show no difference in diversity between d7 and d0 activated cells (cells “not driving” a change in clonality after vaccination). cTfh cells labelled as “driving” clonality were conservatively assigned; all cTfh cells present at the frequency where the diversity of d7 crossed that of d0 were classified as “not driving” clonality.

We utilized DeepTCR (30), a python package (Python v3.10.5) that has a collection of unsupervised and supervised deep learning methods to parse TCRSeq data, to explore the CDR3β repertoire of subject 6. As this methodology has been utilized to isolate antigen-specific TCRs (30), we sought to identify antigen targets of expanded cTfh cell clonotypes. The DeepTCR variational autoencoder was trained using the unsupervised method on the CDR3β sequences of interest using a minimum reconstruction accuracy of 0.9 (accuracy_min) and seed 123 for graph weight initialization (graph_seed). Following training, the 256-dimensional latent space was extracted. Principal Component Analysis (PCA) was run on this learned embedding and the cumulative explained variance ratio was plotted. Cumulative variance was observed to converge within the first 30 principal components (PCs). Uniform Manifold Approximation and Projection (UMAP) was then run on the identified PCs. Initially, all CDR3β sequences from subject 6 were used for training. The resulting UMAP distribution was observed to be driven to a degree by CDR3β length (**Supplementary Figure 5**). For this reason, the analysis was repeated on only the dominant length CDR3β sequences (15 amino acid residues) from subject 6. The resulting UMAP produced distinct islands. Mapping of expanded clonotypes in both d0 resting (**Figure 5D**) and d7 active populations (**Figure 5E**) revealed they were present in multiple islands and at low frequency in the resting population prior to expansion post-vaccination. These results suggest that expanded TCR clonotypes may recognize multiple vaccine antigens.

### Bioinformatics and statistics

Graphpad Prism 8 was used for statistical analyses for **Figures 1**–**4**, and **Supplementary Figures 1, 2**. RStudio Version 1.4.1103 for Mac was used for statistical analyses for **Figures 5**, **6**, and **Supplementary Figures 4, 7** (62). A statistical significance threshold was set at α=0.05 with single cell analyses performed on normalized gene expression counts.

TCR percentages were plotted as a heatmap using “ComplexHeatmap” v2.6.2 in R. Differential gene expression analysis was conducted using “Seurat” v4.0.3 (64–67). The default settings (Wilcoxon Rank Sum test) with min.pct=0 and logfc.threshold=0 were used. Visualisation of DEGs was through the R “EnhancedVolcano” v1.8.0 package (69) with significance set at p < 0.05 and a fold change > |1.5|. Pathway analyses were conducted in R using “clusterProfiler” v3.18.1 (70) for the Gene Ontology (GO) overrepresentation method (33, 34). All GO pathways were analysed. Genes were annotated with GO terms using the “AnnotationDbi” v1.54.1 package in R (71). All genes above a fold change of |1.5| based off previous differential gene expression analyses were used for GO analysis, separated into two groups by sign (positive or negative). Default settings were used with a pvalueCutoff=0.05. Significant pathways were plotted using the package “ggplot2” v3.3.5. Ingenuity pathways analyses (37) were utilized for upstream regulator analyses on significant DEGs identified between resting and expanded cTfh cells (log_2_FC > |1| and p < 0.05). Activated or inhibited upstream drivers were identified by a z-score of > |2|.

## Data availability statement

The datasets presented in this study can be found in online repositories. The names of the repository/repositories and accession number(s) can be found below: PRJNA928207 (SRA).

## Ethics statement

The studies involving human participants were reviewed and approved by Vanderbilt Investigational Review Board. The patients/participants provided their written informed consent to participate in this study.

## Author contributions

JC contributed to designing research studies, conducting experiments, acquiring data, analyzing data, and writing the manuscript. JS contributed to conducting experiments, acquiring data, analyzing data, and editing the manuscript. JO contributed to conducting experiments, acquiring data, analyzing data, and editing the manuscript. SG contributed to designing research studies, analyzing data, and editing the manuscript. CW contributed to conducting experiments, and acquiring data. RG contributed to conducting experiments, and acquiring data. EA contributed to analyzing data RR contributed to acquiring and analyzing data. SL contributed to acquiring and analyzing data. JA contributed to analyzing data and editing the manuscript. RS contributed to sample collection, sample storage, and conducting experiments. AC contributed to acquiring data. NBH developed informed consent documents, contributed to cohort design, oversaw study participant recruitment, and arranged study visit schedules. MP contributed to conceptualization, cohort development, designing research studies, and conducting experiments. SK contributed to conceptualization, cohort development, designing research studies, and editing the manuscript. All authors contributed to the article and approved the submitted version.
