# Supplementary material for: Tracking of activated cTfh cells following sequential influenza vaccinations reveals transcriptional profile of clonotypes driving a vaccine-induced immune response
Source: Front Immunol. 2023 Mar 29;14:1133781. doi: 10.3389/fimmu.2023.1133781 (PMC10095155; doi:10.3389/fimmu.2023.1133781)
Supplement: Supplementary file 1 [file DataSheet_1.pdf]

SUPPLEMENTARY MATERIAL

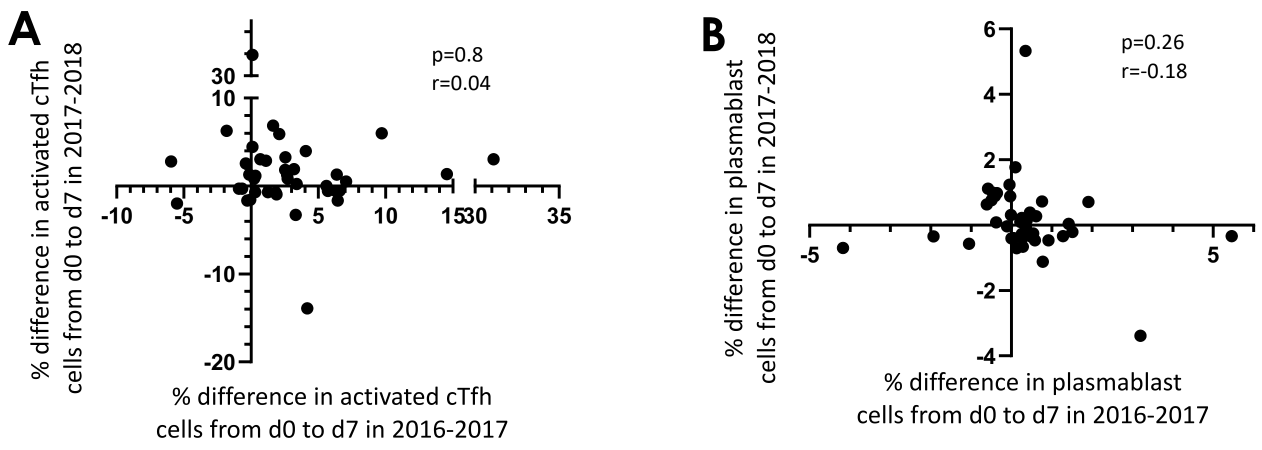

Supplementary Fig 1. The magnitude of **(A)** cTfh and **(B)** plasmablast activation did not correlate between vaccination years.

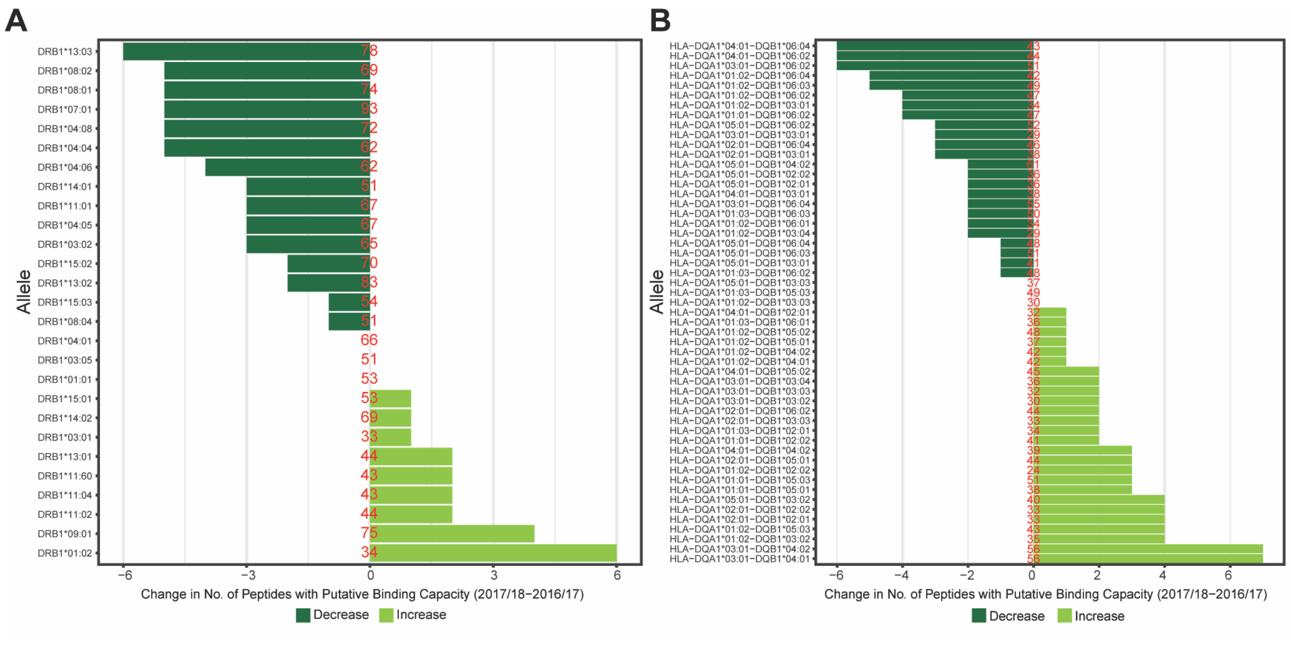

Supplementary Fig 2. Genetic variation in H1N1 strains results in differing numbers of predicted peptides with binding affinities. Peptides with predicted capacity to bind to all HLA-DR alleles **(A)** and -DQ allele combinations **(B)** within the cohort were identified. Using the 2016-17 vaccine season as a baseline, a change in the number of peptides with predicted binding capacity was identified with overlap indicated centrally in red.

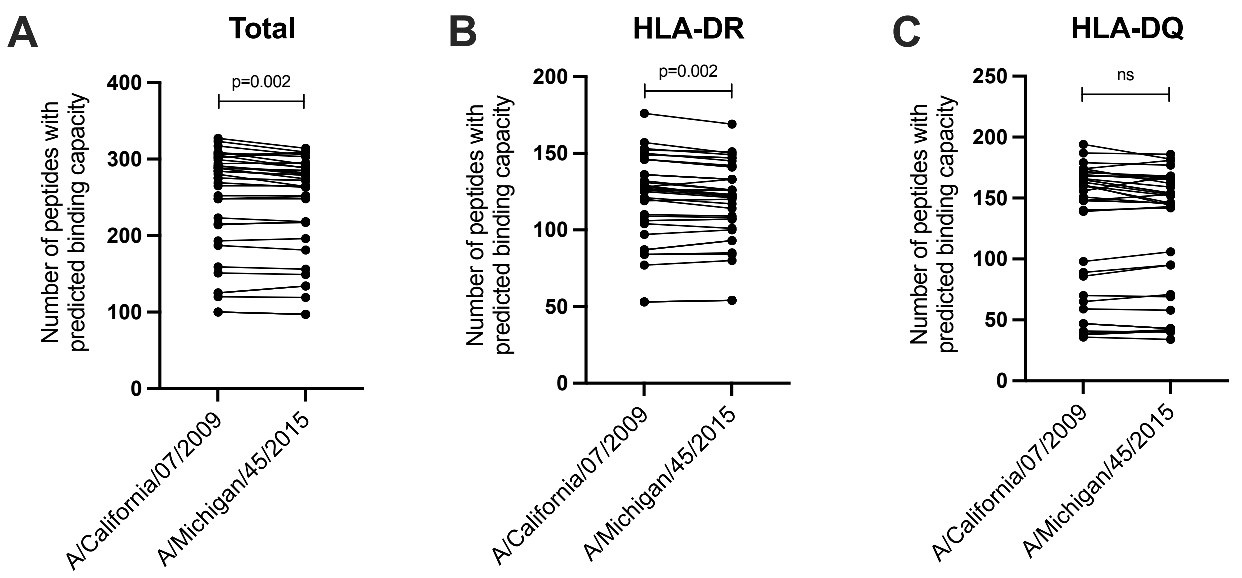

Supplementary Fig 3. Genetic variation between influenza A H1N1 A/California/7/2009 and A/Michigan/45/2015 strains results in differences in predicted peptide binding affinities, potentially impacting subject-specific epitope repertoire. Peptides with predicted capacity to bind to all HLA-DR alleles and allele combinations for each subject were identified. **(A)** Combined counts of predicted HLA-DR/DQ binding peptides shows a small, yet significant, reduction in the 2017-18 season, compared to 2016-17 in this cohort (p = 0.002; Wilcoxon signed rank test). Moreover, this appears to be primarily driven by peptides associated with HLA-DR **(B**; p=0.002; Wilcoxon signed rank test**)**, rather than with HLA-DQ **(C**; p = 0.15; Wilcoxon signed rank test**)**.

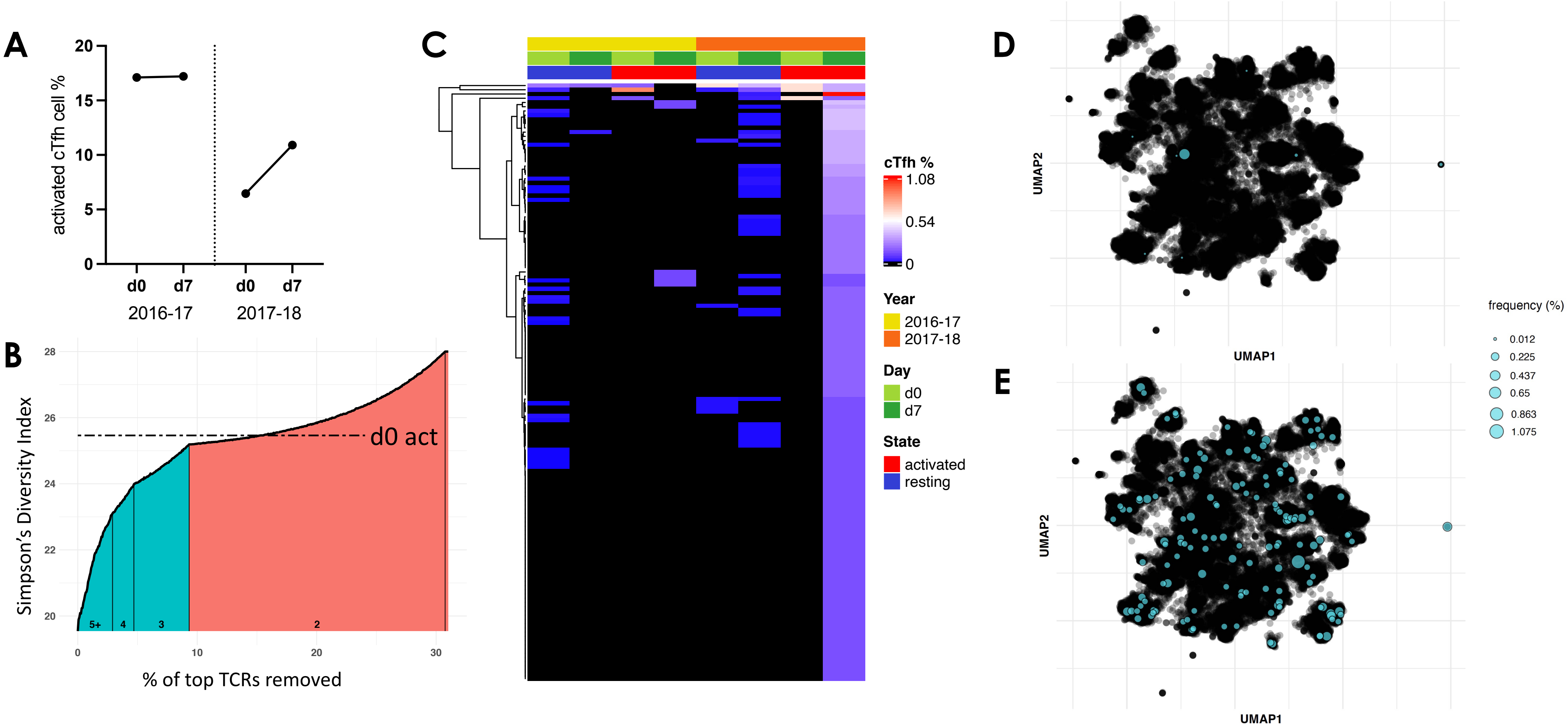

Supplementary Fig 4. A sub-population of cTfh cells is expanded following influenza vaccination in 2017-18. (A) Subject 10 showed an increase in cTfh cell response after the 2017-18 vaccine, with a minimal response the previous season. **(B)** Approximately 10% of activated cTfh cells at d7 expanded based on their TCR repertoire from d0 to d7 following the 2017-18 vaccine (shaded teal). As the intersection with d0 activated cTfh cell diversity fell within a count bracket, TCRs observed at this count (of 2) were not included in those deemed to be expanded. **(C)** The frequencies of cTfh cells with a TCR designated to be expanded from (B) are depicted for all conditions as a heatmap (hierarchical clustering). Of the cells expanded at d7 following the 2017-18 vaccination, very few TCRs were observed following the 2016-17 vaccine, nor in the d0 active populations. The TCR repertoire was clustered and expanded clonotypes post-vaccination in 2017-18 were highlighted for **(D)** d0 resting and **(E)** d7 active conditions.

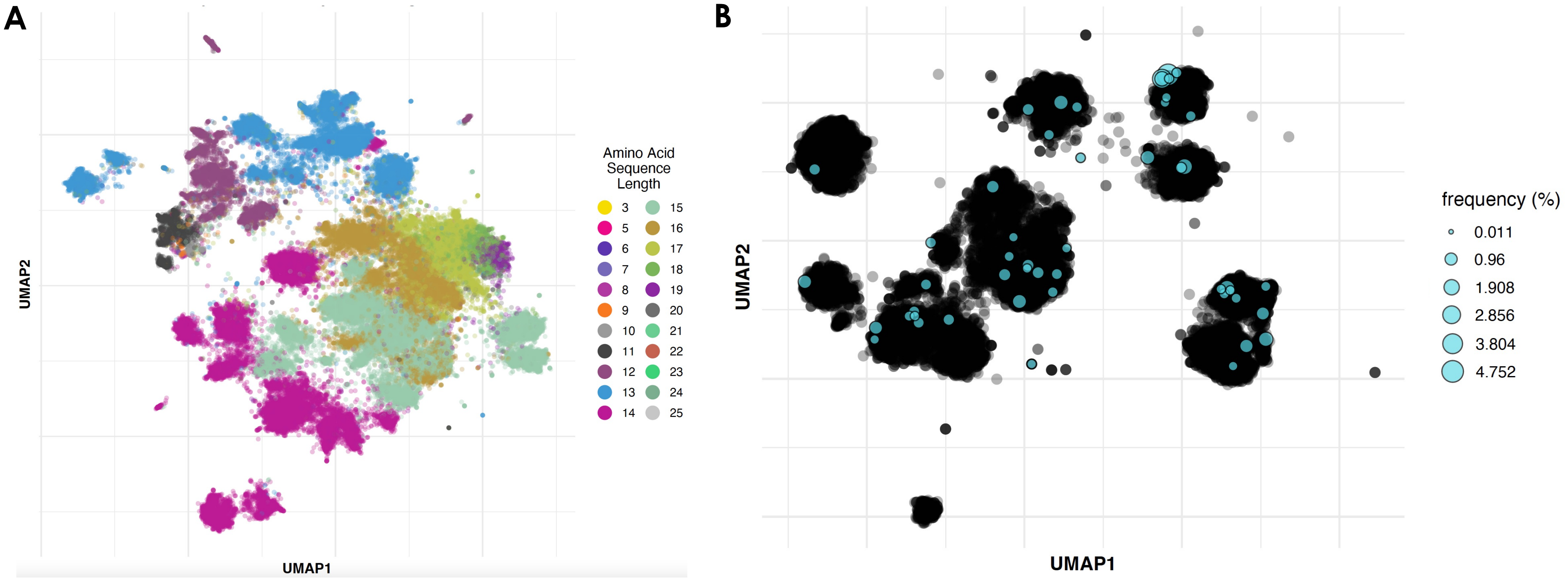

Supplementary Fig 5. TCRs of the same length largely cluster together and expanded clonotypes likely identify different antigens. (A) Clustering of all subject 6 TCRs is largely driven by CDR3 length. **(B)** Using only the dominant CDR3 length of 15, a further sub-clustering was identified. Highlighting those TCRs expanded in the d7 active population in 2016-17 identifies their presence in nearly all clusters.

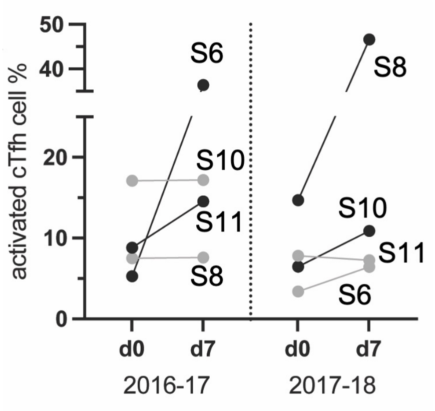

Supplementary Fig 6. Expanded activated cTfh cells sorted for TCRβ sequencing and transcriptomic analysis. Percentage of cTfh cells activated at d0 and d7 for the four subjects selected for single cell sorting. The sorted samples from a season for each subject is in black, with the non-sorted samples from the alternate season in grey.

A

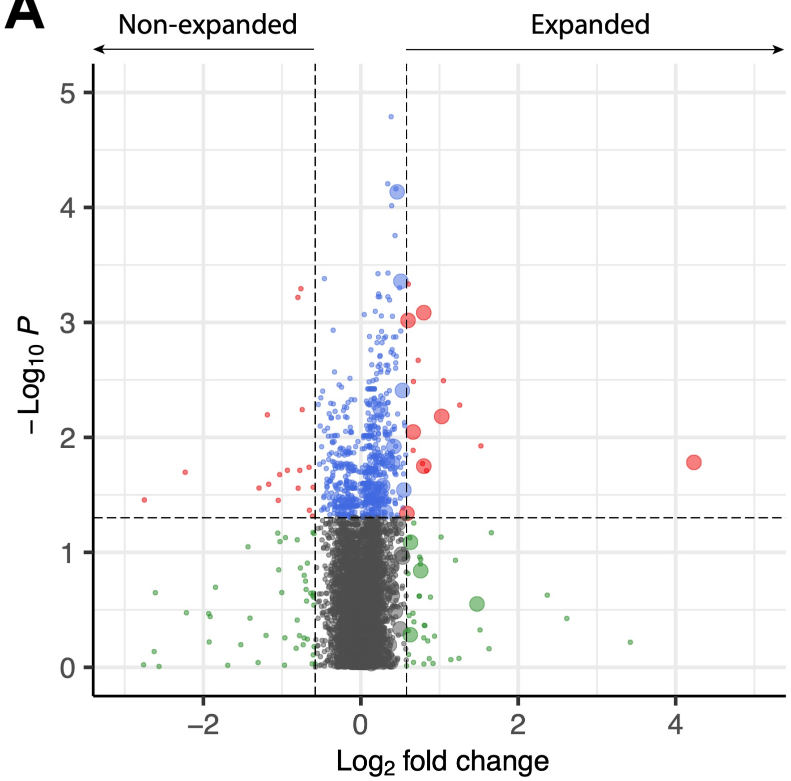

B

Gene	P value	Log2FC
PSMD13	0.001	0.800
ZNF615	0.001	0.600
ARHGEF4	0.007	1.027
MPHOSPH6	0.009	0.664
AHNAK2	0.016	4.232
OSER1	0.018	0.800
MAF	0.046	0.585
SLC7A6	0.082	0.632
SP100	0.144	0.760
IGLV2-8	0.281	1.476
MRPL27	0.521	0.627
MACF1	0.006	0.252
PCDHB4	0.026	0.277
CYTOR	0.045	0.312
PTPRC	0.016	0.340
AP1G1	0.017	0.405
ATP5I	0.012	0.420
RMND5A	0.000	0.462
DCTN5	0.000	0.509
RAC2	0.004	0.528
HIST1H1E	0.029	0.547

C

Gene	P value	Log2FC
RHOG	0.056	0.479
GPI	0.064	0.380
POLR1D	0.104	0.519
CDC42SE1	0.110	0.532
BAZ1A	0.132	0.395
CFD	0.202	0.390
STMN1	0.237	0.400
BAMBI	0.245	-0.295
PARP14	0.269	0.144
ACTG1	0.299	0.005
PTPN7	0.328	0.439
ERAP2	0.400	0.052
ELMO2	0.462	0.499
ISG15	0.505	-0.188
RBM39	0.564	0.095
HMGB3	0.574	0.228
UCP2	0.638	0.362
ANP32B	0.780	-0.030
ITGAL	0.856	0.292
KIAA1429	0.898	0.382
RBM25	0.909	0.047
PSMA4	0.928	0.132

Supplementary Fig 7. Different transcriptional profile between non-expanded and expanded activated cTfh cells at d7. (A) Volcano plot comparing non-expanded and expanded activated cTfh cells for all four subjects at d7. Significant DEGs by both p value and fold change are depicted in red. Horizontal and vertical dotted lines represent a significance threshold of $p < 0.05$ and a fold change of |1.5|, respectively. Significant DEGs identified to be upregulated in expanded cTfh cells compared to resting (Fig 6A) are depicted by an increased point size. Such genes that remained significant by either p value and fold change or by one alone are listed in **(B)**, with those not significant listed in **(C)**.

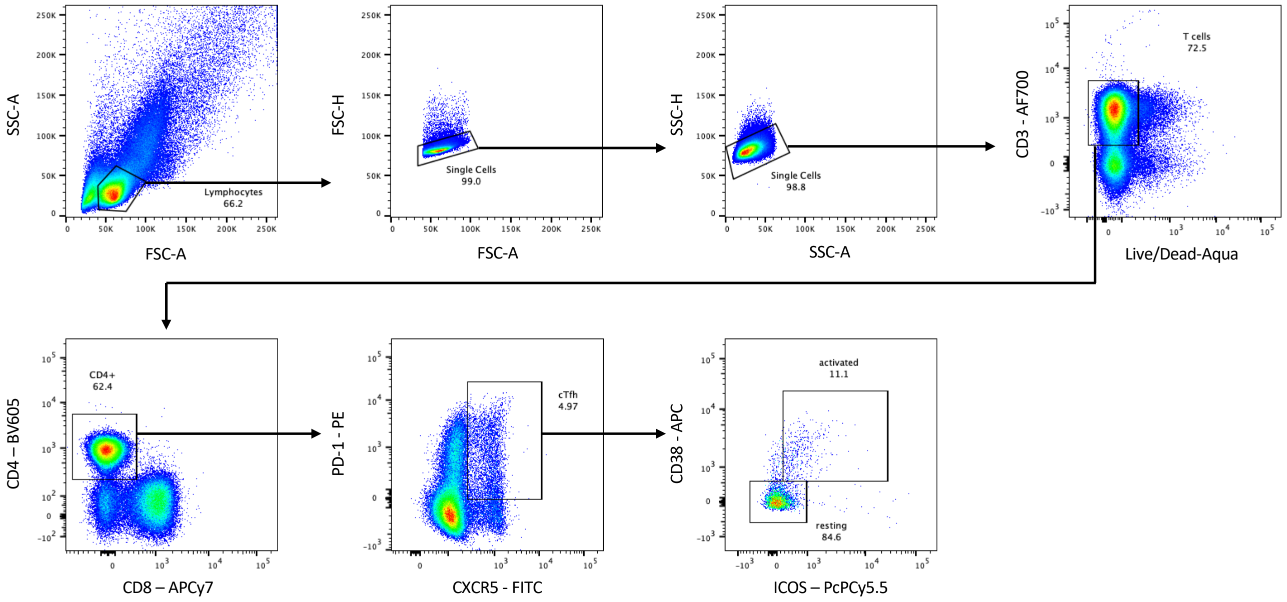

Supplementary Fig 8. Example representation of flow gating for sorting resting and activated cTfh cells. Gating strategy for quantifying and sorting activated and resting cTfh cell populations. The gating strategy was the same for all subjects.

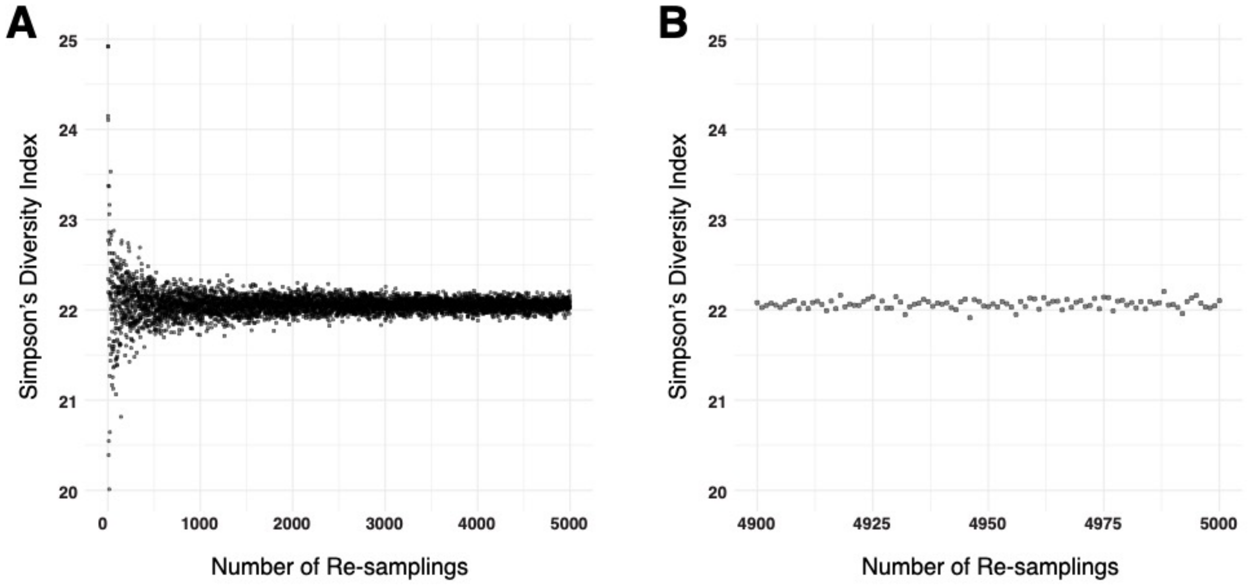

Supplementary Fig 9. Representative plots showing the convergence of the Inverse Simpson's Index after 5000 re-samplings. (A) The Inverse Simpson's Index scores for subject 9 for the 2017-18 season d0 resting cTfh population plotted after each resampling. **(B)** The final 100 re-samplings to show the convergence of scores after 5000 re-samplings.

Supplementary Table 1. Significant DEGs for resting (pct.2) versus expanded (pct.1) cTfh cells.

Gene	p value	avg_log2FC	pct.1	pct.2	p value adj
MACF1	0.0000000079	0.6208870330	0.551	0.304	0.000143
CYTOR	0.0000000090	0.6716239823	0.176	0.033	0.000164
HIST1H1E	0.0000000188	1.1318965066	0.324	0.128	0.000343
ACTG1	0.0000000258	0.6378779815	0.653	0.426	0.000470
PTPRC	0.0000029895	0.7058497206	0.792	0.629	0.054507
UCP2	0.0000073690	0.8315937769	0.366	0.198	0.134359
STMN1	0.0000087225	1.0368868685	0.111	0.021	0.159037
ATP5I	0.0000126579	0.6535103202	0.431	0.252	0.230791
DCTN5	0.0000161529	0.6087335093	0.199	0.076	0.294515
GPI	0.0000177911	0.7981668470	0.301	0.152	0.324384
RBM25	0.0000244956	0.6737479913	0.495	0.316	0.446629
RMND5A	0.0000263174	0.7337272221	0.384	0.237	0.479846
RAC2	0.0000296488	0.7546608568	0.421	0.243	0.540586
PARP14	0.0000364068	0.6573188668	0.269	0.131	0.663805
ELMO2	0.0000394611	1.0133022184	0.083	0.012	0.719495
BAZ1A	0.0000781682	0.6488328349	0.241	0.112	1.000000
PSMA4	0.0000814762	0.6460279037	0.208	0.088	1.000000
RHOG	0.0002819918	0.5875045876	0.269	0.143	1.000000
ANP32B	0.0006924191	0.6292760648	0.417	0.271	1.000000
AP1G1	0.0009377339	0.6063159537	0.398	0.271	1.000000
BAMBI	0.0011299783	0.9619940041	0.176	0.082	1.000000
CFD	0.0014171947	0.5985408109	0.13	0.055	1.000000
MRPL27	0.0018071849	0.7758590696	0.12	0.049	1.000000
PTPN7	0.0018149946	0.6566742544	0.088	0.027	1.000000
CDC42SE1	0.0023083782	0.7943541627	0.282	0.17	1.000000
RBM39	0.0029178671	0.5803056132	0.514	0.386	1.000000
MPHOSPH6	0.0056297656	0.6990185726	0.083	0.03	1.000000
PSMD13	0.0073109283	0.7161037207	0.134	0.067	1.000000
PCDHB4	0.0074239575	0.7315157913	0.25	0.152	1.000000
POLR1D	0.0074988640	0.6580319138	0.148	0.079	1.000000
MAF	0.0076830342	0.5932827616	0.301	0.207	1.000000
ISG15	0.0081573236	0.6602957888	0.218	0.134	1.000000
SP100	0.0093856785	0.7821620123	0.315	0.222	1.000000
IGLV2-8	0.0133890338	1.5251779139	0.019	0	1.000000
ZNF615	0.0137175731	0.6249298878	0.046	0.012	1.000000
HMGB3	0.0139987762	0.7576071325	0.292	0.207	1.000000
AHNAK2	0.0181253260	0.8897758147	0.056	0.018	1.000000
KIAA1429	0.0205917666	0.6541444338	0.144	0.082	1.000000
ERAP2	0.0235117779	0.7446093114	0.134	0.076	1.000000
SLC7A6	0.0273358181	0.7511199994	0.13	0.073	1.000000
ARHGEF4	0.0276064141	0.7382693346	0.042	0.012	1.000000
OSER1	0.0291831097	0.7157346527	0.102	0.052	1.000000
ITGAL	0.0329792909	0.5961151515	0.208	0.149	1.000000
HBA2	0.0000000001	-2.5203744888	0	0.173	0.000002
CXCR4	0.0000000013	-1.5938117806	0.218	0.456	0.000024
TXNIP	0.0000012837	-1.0370515296	0.514	0.653	0.023405
IL7R	0.0011426220	-0.5862386199	0.505	0.629	1.000000
SERPINB9	0.0022591109	-0.5963638969	0.111	0.204	1.000000
HBA1	0.0026303910	-0.8667824357	0.005	0.052	1.000000
HK3	0.0036269135	-2.8547663473	0.505	0.584	1.000000
EEF1B2	0.0050037217	-0.6016907093	0.407	0.477	1.000000
HBB	0.0057064382	-0.7846702840	0.005	0.046	1.000000
ACTR3C	0.0059350225	-0.6729292926	0.069	0.143	1.000000
STOX1	0.0116885829	-1.2677958807	0.019	0.064	1.000000
CLOCK	0.0286693031	-0.6451162131	0.259	0.173	1.000000
AHNAK	0.0301148848	-0.7498502452	0.509	0.562	1.000000
PFDN5	0.0311793671	-0.6045528229	0.63	0.66	1.000000
IGKC	0.0385136137	-1.2523270923	0.111	0.173	1.000000

Supplementary Table 2. Significant DEGs for non-expanded (pct.2) versus expanded (pct.1) activated cTfh cells.

Gene	p value	avg_log2FC	pct.1	pct.2	pvalue adj
SELPLG	0.000464212	0.6032718	0.389	0.261	1
PSMD13	0.00082306	0.79966	0.134	0.056	1
ZNF615	0.000960858	0.5996825	0.046	0.006	1
ABCG4	0.002138128	0.7281582	0.042	0.006	1
SBSN	0.003211934	1.049395	0.032	0.003	1
CTD-2503O16.4	0.003265329	0.668588	0.032	0.003	1
C8orf46	0.005243575	1.2560952	0.148	0.075	1
ARHGEF4	0.006578019	1.0273812	0.042	0.008	1
MPHOSPH6	0.008972941	0.6644007	0.083	0.033	1
SNTB2	0.011871904	1.5255788	0.278	0.192	1
TP53INP1	0.012981393	0.6648019	0.12	0.061	1
AHNAK2	0.016496905	4.2318998	0.056	0.019	1
CRB2	0.016886146	0.7845745	0.046	0.014	1
OSER1	0.017789568	0.8000804	0.102	0.05	1
FAM120B	0.019493493	0.8340513	0.259	0.181	1
MAF	0.045887326	0.5853296	0.301	0.239	1
FAM227A	0.0005093	-0.7616489	0.171	0.289	1
EGFR	0.000605735	-0.799702	0	0.053	1
ZNF267	0.005739967	-0.744954	0.056	0.125	1
NR2F1	0.006363269	-1.1874837	0.028	0.083	1
OR2C3	0.018217526	-0.6576589	0.292	0.364	1
RP11-344P13.4	0.019344393	-0.774931	0	0.025	1
SPATA31E1	0.019344393	-0.9330313	0	0.025	1
STOX1	0.020125014	-2.2308595	0.019	0.058	1
ZNF773	0.021086585	-1.0311055	0.019	0.058	1
ZNF469	0.025595045	-1.1711559	0.009	0.042	1
GPR161	0.027144805	-0.6049876	0.023	0.064	1
PON1	0.027594556	-1.2928434	0	0.022	1
NOVA1	0.027647795	-0.7964148	0.347	0.414	1
LGI2	0.035010374	-2.7498361	0.009	0.039	1
HDAC4	0.035321933	-1.0489471	0.046	0.092	1
ACTR3C	0.043041899	-0.6534583	0.069	0.119	1
USP45	0.048388466	-0.6150227	0.023	0.058	1

Supplementary Table 3. Significant genes upregulated in GO pathways associated with resting cTfh cells.

Pathway	pvalue	padjust	Genes	DEGs
response to hydrogen peroxide	2.6E-06	1.9E-03	TNFAIP3/JUN/HBB/HBA1/TXNIP/HBA2	6
response to reactive oxygen species	2.6E-06	1.9E-03	TNFAIP3/JUN/HBB/HBA1/APOE/TXNIP/HBA2	7
response to oxidative stress	2.5E-05	1.2E-02	TNFAIP3/JUN/HBB/HBA1/APOE/TXNIP/STOX1/HBA2	8
hydrogen peroxide catabolic process	3.6E-05	1.3E-02	HBB/HBA1/HBA2	3
cellular oxidant detoxification	9.3E-05	2.7E-02	HBB/HBA1/APOE/HBA2	4
cellular detoxification	1.5E-04	3.3E-02	HBB/HBA1/APOE/HBA2	4
bicarbonate transport	1.7E-04	3.3E-02	HBB/HBA1/HBA2	3
cellular response to toxic substance	1.8E-04	3.3E-02	HBB/HBA1/APOE/HBA2	4
response to mechanical stimulus	2.4E-04	3.4E-02	JUN/HDAC4/TXNIP/CXCR4/CNTNAP2	5
hydrogen peroxide metabolic process	2.6E-04	3.4E-02	HBB/HBA1/HBA2	3
detoxification	2.6E-04	3.4E-02	HBB/HBA1/APOE/HBA2	4
endocytic vesicle lumen	1.7E-07	2.4E-05	HBB/HBA1/APOE/HBA2	4
blood microparticle	9.0E-06	6.5E-04	HBB/HBA1/APOE/IGKC/HBA2	5
cytosolic small ribosomal subunit	6.6E-04	3.2E-02	HBA1/HBA2	2
oxygen binding	8.8E-06	1.8E-03	HBB/HBA1/HBA2	3
antioxidant activity	3.5E-05	3.6E-03	HBB/HBA1/APOE/HBA2	4
peroxidase activity	2.1E-04	1.4E-02	HBB/HBA1/HBA2	3
oxidoreductase activity, acting on peroxide as acceptor	2.8E-04	1.4E-02	HBB/HBA1/HBA2	3

Supplementary Table 4. Significant genes upregulated in GO pathways associated with expanded cTfh cells.

Pathway	pvalue	padjust	Genes	DEGs
Fc receptor signaling pathway	3.3E-10	4.2E-07	IGLC2/IGLV2-8/IGHG3/ELMO2/IGLV2-14/IGLC3/PSMD13/PTPRC/GRAP2/PSMA4/ACTG1	11
phagocytosis	7.2E-10	4.5E-07	IGLC2/IGLV2-8/IGHG3/ELMO2/IGLV2-14/IGLC3/CDC42SE1/RAC2/PTPRC/ACTG1/ITGAL/RHOG	12
immune response-regulating cell surface receptor signaling pathway involved in phagocytosis	4.3E-09	1.3E-06	IGLC2/IGLV2-8/IGHG3/ELMO2/IGLV2-14/IGLC3/PTPRC/ACTG1	8
Fc-gamma receptor signaling pathway involved in phagocytosis	4.3E-09	1.3E-06	IGLC2/IGLV2-8/IGHG3/ELMO2/IGLV2-14/IGLC3/PTPRC/ACTG1	8
Fc-gamma receptor signaling pathway	5.5E-09	1.4E-06	IGLC2/IGLV2-8/IGHG3/ELMO2/IGLV2-14/IGLC3/PTPRC/ACTG1	8
Fc receptor mediated stimulatory signaling pathway	7.1E-09	1.5E-06	IGLC2/IGLV2-8/IGHG3/ELMO2/IGLV2-14/IGLC3/PTPRC/ACTG1	8
activation of immune response	1.3E-07	2.3E-05	IGLC2/IGLV2-8/IGHG3/ELMO2/IGLV2-14/IGLC3/PSMD13/PTPRC/GRAP2/PSMA4/ACTG1/CFD	12
immune response-activating cell surface receptor signaling pathway	2.3E-07	3.2E-05	IGLC2/IGLV2-8/IGHG3/ELMO2/IGLV2-14/IGLC3/PSMD13/PTPRC/GRAP2/PSMA4/ACTG1	11
immune response-activating signal transduction	2.3E-07	3.2E-05	IGLC2/IGLV2-8/IGHG3/ELMO2/IGLV2-14/IGLC3/PSMD13/PTPRC/GRAP2/PSMA4/ACTG1	11
humoral immune response mediated by circulating immunoglobulin	2.8E-07	3.2E-05	IGLC2/IGLV2-8/IGHG3/IGLV2-14/IGLC3/PTPRC	6
regulation of humoral immune response	2.8E-07	3.2E-05	IGLC2/IGLV2-8/IGHG3/IGLV2-14/IGLC3/PTPRC	6
complement activation	4.7E-07	4.9E-05	IGLC2/IGLV2-8/IGHG3/IGLV2-14/IGLC3/CFD	6
immune response-regulating cell surface receptor signaling pathway	5.6E-07	5.2E-05	IGLC2/IGLV2-8/IGHG3/ELMO2/IGLV2-14/IGLC3/PSMD13/PTPRC/GRAP2/PSMA4/ACTG1	11
immune response-regulating signaling pathway	6.0E-07	5.2E-05	IGLC2/IGLV2-8/IGHG3/ELMO2/IGLV2-14/IGLC3/PSMD13/PTPRC/GRAP2/PSMA4/ACTG1	11
humoral immune response	6.3E-07	5.2E-05	IGLC2/IGLV2-8/IGHG3/IGLV2-14/IGLC3/GPI/PTPRC/CFD	8
Fc-epsilon receptor signaling pathway	7.4E-07	5.8E-05	IGLC2/IGLV2-8/IGLV2-14/IGLC3/PSMD13/GRAP2/PSMA4	7
complement activation, classical pathway	2.4E-06	1.7E-04	IGLC2/IGLV2-8/IGHG3/IGLV2-14/IGLC3	5
regulation of complement activation	2.4E-06	1.7E-04	IGLC2/IGLV2-8/IGHG3/IGLV2-14/IGLC3	5
phagocytosis, engulfment	1.0E-05	6.9E-04	IGLC2/IGHG3/IGLC3/RAC2/RHOG	5
regulation of immune effector process	1.5E-05	9.2E-04	IGLC2/IGLV2-8/IGHG3/IGLV2-14/IGLC3/GPI/RAC2/PTPRC/AP1G1	9
immunoglobulin mediated immune response	1.6E-05	9.3E-04	IGLC2/IGLV2-8/IGHG3/IGLV2-14/IGLC3/PTPRC	6
B cell mediated immunity	1.7E-05	9.7E-04	IGLC2/IGLV2-8/IGHG3/IGLV2-14/IGLC3/PTPRC	6
plasma membrane invagination	1.9E-05	1.0E-03	IGLC2/IGHG3/IGLC3/RAC2/RHOG	5
membrane invagination	3.0E-05	1.6E-03	IGLC2/IGHG3/IGLC3/RAC2/RHOG	5
lymphocyte mediated immunity	7.5E-05	3.7E-03	IGLC2/IGLV2-8/IGHG3/IGLV2-14/IGLC3/PTPRC/AP1G1	7
antigen receptor-mediated signaling pathway	7.7E-05	3.7E-03	IGLC2/IGHG3/IGLC3/PSMD13/PTPRC/GRAP2/PSMA4	7
leukocyte migration	1.4E-04	6.4E-03	IGLC2/IGLV2-8/IGLV2-14/IGLC3/RAC2/SLC7A6/ITGAL/RHOG	8
regulation of B cell activation	1.8E-04	7.9E-03	IGLC2/IGHG3/IGLC3/HMGB3/PTPRC	5
B cell receptor signaling pathway	2.4E-04	1.0E-02	IGLC2/IGHG3/IGLC3/PTPRC	4
regulation of lymphocyte activation	2.5E-04	1.0E-02	IGLC2/IGHG3/IGLC3/HMGB3/RAC2/PTPRC/GRAP2/AP1G1	8
phagocytosis, recognition	2.9E-04	1.2E-02	IGLC2/IGHG3/IGLC3	3
positive regulation of B cell activation	4.5E-04	1.8E-02	IGLC2/IGHG3/IGLC3/PTPRC	4
adaptive immune response based on somatic recombination of immune receptors built from immunoglobulin superfamily domains	6.7E-04	2.5E-02	IGLC2/IGLV2-8/IGHG3/IGLV2-14/IGLC3/PTPRC	6
regulation of leukocyte activation	7.2E-04	2.6E-02	IGLC2/IGHG3/IGLC3/HMGB3/RAC2/PTPRC/GRAP2/AP1G1	8
leukocyte degranulation	7.6E-04	2.7E-02	GPI/RAC2/PSMD13/PTPRC/AP1G1/CFD/ITGAL/RHOG	8
antigen processing and presentation of peptide antigen	8.6E-04	3.0E-02	ERAP2/PSMD13/PSMA4/DCTN5/AP1G1	5
positive regulation of lymphocyte activation	9.0E-04	3.1E-02	IGLC2/IGHG3/IGLC3/PTPRC/GRAP2/AP1G1	6
negative regulation of peptidyl-tyrosine phosphorylation	9.2E-04	3.1E-02	SOCS4/PTPRC/PARP14	3
immunoglobulin production	1.3E-03	4.3E-02	IGLV2-8/IGLV2-14/GPI/PTPRC	4
immunoglobulin complex	1.9E-07	3.7E-05	IGLC2/IGLV2-8/IGHG3/IGLV2-14/IGLC3	5
immunoglobulin complex, circulating	8.6E-05	8.2E-03	IGLC2/IGHG3/IGLC3	3
blood microparticle	4.9E-04	3.1E-02	IGLC2/IGHG3/IGLC3/ACTG1	4
antigen binding	1.9E-05	3.6E-03	IGLC2/IGLV2-8/IGHG3/IGLV2-14/IGLC3	5
immunoglobulin receptor binding	1.3E-04	1.2E-02	IGLC2/IGHG3/IGLC3	3

Supplementary Table 5. The number of unique and total CDR3s identified for each subject by condition.

Subject	Year	Day	State	No. unique CDR3s	Total CDR3s
2	2016-17	d0	activated	360	481
			resting	6016	8186
		d7	activated	40	50
			resting	66	71
	2017-18	d0	activated	28	34
			resting	299	381
		d7	activated	174	223
			resting	3001	4148
3	2016-17	d0	activated	93	112
			resting	2005	2815
		d7	activated	287	387
			resting	1909	2511
	2017-18	d0	activated	132	161
			resting	2219	2942
		d7	activated	29	37
			resting	350	476
4	2016-17	d0	activated	134	159
			resting	5587	7835
		d7	activated	532	784
			resting	6987	9963
	2017-18	d0	activated	573	731
			resting	10728	16717
		d7	activated	186	202
			resting	5607	7984
5	2016-17	d0	activated	1133	1332
			resting	22715	30442
		d7	activated	4675	9327
			resting	21034	27635
	2017-18	d0	activated	2089	2539
			resting	26777	37528
		d7	activated	1385	1619
			resting	17742	23698
6	2016-17	d0	activated	628	719
			resting	25431	36637
		d7	activated	1810	4056
			resting	14879	20002
	2017-18	d0	activated	291	327
			resting	17152	24280
		d7	activated	492	543
			resting	11023	14645
7	2016-17	d0	activated	195	225
			resting	4222	5710
		d7	activated	1806	2710
			resting	4960	6859
	2017-18	d0	activated	0	0
			resting	19258	27391
		d7	activated	1186	1681
			resting	2535	3614

Subject	Year	Day	State	No. unique CDR3s	Total CDR3s
8	2016-17	d0	activated	101	119
			resting	1170	1447
		d7	activated	137	158
			resting	2732	3311
	2017-18	d0	activated	129	139
			resting	4591	5742
		d7	activated	555	812
			resting	3133	4147
9	2016-17	d0	activated	498	668
			resting	25941	38598
		d7	activated	3355	5479
			resting	36368	53693
	2017-18	d0	activated	890	1204
			resting	38586	57739
		d7	activated	858	1227
			resting	36564	54700
10	2016-17	d0	activated	3014	6088
			resting	17870	23546
		d7	activated	667	888
			resting	3207	3849
	2017-18	d0	activated	273	319
			resting	6637	8253
		d7	activated	1512	2325
			resting	9436	11724
11	2016-17	d0	activated	575	655
			resting	9855	12698
		d7	activated	872	1079
			resting	6229	8039
	2017-18	d0	activated	1067	1332
			resting	8472	11121
		d7	activated	1520	1937
			resting	3950	4508
12	2016-17	d0	activated	1328	1897
			resting	16699	24959
		d7	activated	177	220
			resting	29178	41424
	2017-18	d0	activated	2237	3629
			resting	7267	9970
		d7	activated	3632	5792
			resting	20562	27912
14	2016-17	d0	activated	32	36
			resting	3122	4286
		d7	activated	578	737
			resting	1187	1486
	2017-18	d0	activated	800	956
			resting	8265	12977
		d7	activated	589	780
			resting	4782	6289

Supplementary Table 6. Median read count (interquartile range) and average per well by subject included for RNAseq analysis for resting and activated cTfh cells.

Subject	Year	State	Median (IQR)	Average
6	2016-17	resting	4221 (2387-6863)	5219
		activated	3210 (1960-7066)	4796
8	2017-18	resting	3272 (1611-5700)	4259
		activated	2756 (1793-3708)	3005
10	2017-18	resting	2092 (1204-3558)	2691
		activated	2498 (1322-3433)	2574
11	2016-17	resting	1881 (1164-2889)	2689
		activated	2640 (1345-4191)	3418
